# Development and clinical validation of a microfluidic-based platform for CTC enrichment and downstream molecular analysis

**DOI:** 10.3389/fonc.2023.1238332

**Published:** 2023-10-02

**Authors:** Songhua Cai, Youjun Deng, Zhe Wang, Junyu Zhu, Chujian Huang, Longde Du, Chunguang Wang, Xiangyang Yu, Wenyi Liu, Chenglin Yang, Zhe Wang, Lixu Wang, Kai Ma, Rui Huang, Xiaoyu Zhou, Heng Zou, Wenchong Zhang, Yan Huang, Zhi Li, Tiaoping Qin, Tao Xu, Xiaotong Guo, Zhentao Yu

**Affiliations:** ^1^ Department of Thoracic Surgery, National Cancer Center/National Clinical Research Center for Cancer/Cancer Hospital and Shenzhen Hospital, Chinese Academy of Medical Sciences and Peking Union Medical College, Shenzhen, China; ^2^ Department of Oncology, The First Affiliated Hospital of Guangdong Pharmaceutical University, Guangzhou, China; ^3^ Institute of Cancer Control, Cancer Hospital of Xinjiang Medical University, Urumqi, China; ^4^ Shenzhen Futian Research Institute, City University of Hong Kong, Shenzhen, China; ^5^ Department of Medical Affairs, Cellomics (ShenZhen) Limited, Shenzhen, China

**Keywords:** microfluidic chip, circulating tumor cells, CTC separation platforms, downstream molecular analysis, CTC counts

## Abstract

**Background:**

Although many CTC isolation and detection methods can provide information on cancer cell counts, downstream gene and protein analysis remain incomplete. Therefore, it is crucial to develop a technology that can provide comprehensive information on both the number and profile of CTC.

**Methods:**

In this study, we developed a novel microfluidics-based CTC separation and enrichment platform that provided detailed information about CTC.

**Results:**

This platform exhibits exceptional functionality, achieving high rates of CTC recovery (87.1%) and purification (∼4 log depletion of WBCs), as well as accurate detection (95.10%), providing intact and viable CTCs for downstream analysis. This platform enables successful separation and enrichment of CTCs from a 4 mL whole-blood sample within 15 minutes. Additionally, CTC subtypes, selected protein expression levels on the CTC surface, and target mutations in selected genes can be directly analyzed for clinical utility using immunofluorescence and real-time polymerase chain reaction, and the detected PD-L1 expression in CTCs is consistent with immunohistochemical assay results.

**Conclusion:**

The microfluidic-based CTC enrichment platform and downstream molecular analysis together provide a possible alternative to tissue biopsy for precision cancer management, especially for patients whose tissue biopsies are unavailable.

## 1 Introduction

Advances in cancer diagnosis and therapy have greatly improved the survival rate of cancer patients in the past years. Liquid biopsy is one of the most promising approaches for precision cancer diagnosis and screening. The analytes of liquid biopsy mainly include circulating tumor cells (CTCs), circulating tumor DNA (ctNDA) and exosome. Among the most promising liquid analytes are CTCs, which are rare subsets of malignant cells that shed from primary or metastatic tumors into the peripheral blood circulation of cancer patients. They are one of the most important biomarkers for liquid biopsy, as they convey important information useful for cancer diagnosis, treatment, and prognosis. CTCs play an important role in cancer metastasis, and their count, phenotype, and genetic characteristics carry abundant multidimensional information about the primary tumor. The clinical utility of CTCs holds remarkable potential in improving cancer detection, monitoring, and management (1).

CTC detection techniques with high sensitivity and specificity are critical for clinical application. However, due to the extremely low number of CTCs present in the blood, their detection poses significant challenges. While several CTC detection platforms have been developed in the past decades, but most of them have shown limitations in clinical utility (**Table S1**). Based on the method of CTC identification, these platforms can be categorized as label-free or label-dependent. Label-free platforms separate CTCs from other cells depending on the physical characteristics, that is, through microfiltration, inertial focusing, centrifugal forces, etc. These techniques are advantageous in terms of rapid processing, but the efficiency and purity of the collected CTCs are usually low, which may compromise the downstream analysis. Label-dependent techniques separate CTCs from the other cells depending on the antigen-antibody affinity of specific tumor cell markers and capture the CTCs through a positive selection of the CTCs or negative depletion of white blood cells (2). These techniques are widely used and are advantageous in enriching the high purity of the CTCs. The CellSearch™ system is a good example of a label-dependent strategy that enriches the CTCs by utilizing the epithelial biomarker EpCAM, a surface protein usually expressed on the epithelial cell membrane. However, because of tumor heterogeneity and the occurrence of epithelial-mesenchymal transition (EMT), it is common that large fractions of CTCs in various cancers exhibit low or nil expression of epithelioid markers, which remarkably compromises the clinical utility of isolation based on positive selection of EpCAM-based CTCs. Furthermore, most of the currently available CTC detection platforms (both label-free and label-dependent platforms) can provide information about the number of tumor cells, but downstream gene and protein analysis are not very mature and complete, which hinder the clinical application of CTCs (3–6). This information would also be helpful for clinical diagnosis, disease monitoring, and therapy selection.

Microfluidic-based technology uses microscale channels and structures to enable fluid control and cell manipulation. Microfluidic-based approaches have been proposed for CTC isolation, wherein CTCs are separated from other cells based on antigen-antibody affinity, size difference, and fluid dynamics. Inertial focusing involves applying the effects of fluid inertia in microchannels of a certain shape to separate cells of various sizes and densities at a high flow rate. When randomly dispersed cells flow rapidly in a curved microchannel, they are subjected to several forces, such as shear-induced lift force (FIL), wall-induced lift force (FWL), and drag force of Dean flow. The combination of many forces in a microchannel enables the migration of cells to a different equilibrium position for automatic focusing. The platforms based on inertial focusing are advantageous in terms of being high throughput, having strong maneuverability, and performing enrichment automatically (7). However, they also have shortcomings such as sample blockage, low CTC recovery rate, limited depletion of leukocytes, and poor cell viability (8).

In addition to the enumeration of CTCs, obtaining comprehensive information contained in CTCs from a whole tumor cell holds great potential in precision oncology. The molecular characteristics of CTCs are useful in cancer monitoring and management (9–11). In clinical practice, CTC enumeration and profiling, especially with regard to protein biomarkers and gene mutations, is expected to have increasing importance in improving the treatment design toward personalized medicine (12).

**Figure 1:**
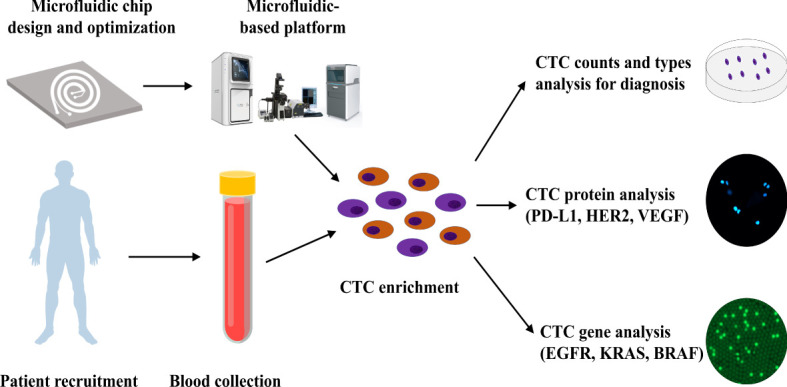
CTC isolation and downstream analysis. Workflow of the microfluidic-based system for CTC isolation and downstream analysis.

In this study, we developed a novel inertial focusing-based CTC platform that utilizes trapezoidal cross-sectional microfluidic channels, wherein CTCs can be isolated from 4 mL whole-blood samples within 15 min. This platform provided in-depth information about CTCs, including tumor cell count, epithelial-mesenchymal transition subtypes, protein expression levels, and target gene mutations. It demonstrated better functionality than the available CTC detection techniques in terms of the high recovery rate (87.1%), high CTC purification rate (∼4 log depletion of white blood cells [WBCs]), and intact viability and integrity of the CTCs. These advantages enable performing protein and gene analysis in a wide range of CTC subsets, independent of cancer cell-specific marker expression. Based on this microfluidics platform, we designed a model for CTC detection and downstream molecular analysis (**Figure 1**), including CTC EMT subtypes identification, target protein analysis, gene mutation analysis, and cell culturing.

The detection and characterization of CTCs that originate from primary and metastatic tumors play an important role in cancer diagnosis, disease monitoring, and prognosis prediction (13). Cancer metastasis occurs when CTCs travel across the body and invade healthy tissues (14). Valuable therapeutic strategies can be applied by monitoring CTCs. Numerous platforms have been developed for the separation of CTCs, including immunomagnetic isolation using antibodies (15), microfiltration (16), and dielectrophoresis (17). However, most of these platforms have various disadvantages, such as high cost, limited sample volume, slow turnaround time, and moderate sensitivity (**Table S1**). There are strong demands for identifying new CTC isolating techniques with increased efficiency and purity outcome but with reduced reagent consumption, sample volume, and analysis time.

## 2 Materials and methods

### 2.1 Fabrication and integration of the microfluidic platform

We designed a mold with specific channel dimensions using SolidWorks software and fabricated it using photolithography technology on a SU8 photoresist for subsequent polydimethylsiloxane (PDMS) casting. The fixative was mixed with Sylgard™ 184 (Dow Corning, USA) at a 1:10 ratio to prepare PDMS glue. Degassed PDMS glue was then poured onto the mold and solidified at 85°C in an oven for 30 min. The PDMS glue was peeled off from the mold, and four fluid channels (two inlets and two outlets) were punched into the solidified mold. A plasma cleaner was used to bond the PDMS glue onto a glass sheet firmly to complete the channels. Finally, the prepared microfluidics chips was placed in an oven and heated at 85°C for 30 min to further enhance the bonding.

### 2.2 Design and optimization of chip parameters

To optimize chip parameters, two types of fluorescent microbeads (polystyrene material; Duke) were used for stimulation: one to simulate the WBCs (9 µm) and the other to stimulate the CTCs (15 µm). One milliliter of 9-µm microbeads (10^6^/mL) was mixed with 1 mL of 15-µm microbeads (10^5^/mL) for the test. To observe the flow traces of these microbeads in the microchannel, the microfluidic chip was mounted on a Nikon inverted microscope, and the buffer inlet and sample inlet were connected to two micro-syringe pumps through a Teflon tube. First, the buffer (1× phosphate-buffered saline [PBS]) was pumped into the sample inlet at a flow rate of 3000 µL/min, followed by the pumping of the sample solution into the sample inlet.

### 2.3 Cell line culture and sample preparation

Human cervical cancer cells (HeLa; CTCC-001-0006, MeisenCTCC), breast cancer cells (SK-BR-3; CTCC-001-0016, MeisenCTCC), lung cancer cells (H1650; CTCC-400–0171, MeisenCTCC), prostate cancer cells (PC-3; CTCC-001-0037, MeisenCTCC), pancreatic cancer cells (PANC-1, CTCC-001-0005, MeisenCTCC), human lung squamous carcinoma cells (H226; CL-0396, Pricella CTCC), human colorectal adenocarcinoma cells (HT-29,CTCC-001-0003, MeisenCTCC), human ductal carcinoma cells (T-47D, CTCC-001-0043, MeisenCTCC), human brest cancer cells (MCF-7, CL-0149, Pricella CTCC) were used to assess the performance of the microfluidic device. HeLa, H1650, PC-3, and PANC-1 cells were cultured in a medium containing 90% Dulbecco's Modified Eagle's Medium (DMEM; GIBCO), 10% fetal bovine serum (FBS), and 1% penicillin/streptomycin. SK-BR-3 cells were cultured in a medium containing 80% DMEM (GIBCO), 20% FBS, 1% L-glutamine, and 1% penicillin/streptomycin. HT-29 cells were cultured in a medium containing 90% RPMI 1640, 10% FBS, 1% penicillin/streptomycin, and 1% L-Glutamine Solution (GLN; BI) and insulin (0.01 mg/mL). T-47D and H226 cells were cultured in a medium containing 90% RPMI 1640, 10% FBS and 1% penicillin/streptomycin. MCF-7 cells were cultured in a medium containing 90% DMEM (GIBCO), 10% FBS, 1% penicillin/streptomycin, 1% L-Glutamine Solution (GLN; BI) and insulin (0.01 mg/mL). All the cultures were maintained in a humidified atmosphere at 37 °C under 5% (v/v) CO2 and harvested at 80% confluence for spiking.

### 2.4 CMFDA (CellTracker™) staining

Sub-confluent monolayers of tumor cells were dissociated by adding 0.01% trypsin and 5.3 mM EDTA solution (Lonza, Switzerland) to the cell culture. The dissociated tumor cell concentration was calculated, and 2×10^5^-5×10^5^ tumor cells were added into 1 mL of RPMI 1640 serum-free medium. Subsequently, 2 µL of CellTracker™ Deep Red Dye (final concentration: 2 µM/µL; Thermo Fisher Scientific, USA) was added, and the cells were stained at 37°C for 20 min.

### 2.5 Cell viability experiments

Approximately 40 SK-BR-3 cells were prepared and spiked into 2 mL of PBS. For the control group, the tumor cells were then transferred into the culture medium (90% McCoy’s 5A, 10% FBS, 1% L-glutamine, and 1% penicillin/streptomycin). For the test group, the tumor cells were processed using the microfluidic detection platform; after processing, the SK-BR-3 cells was collected and added into the same culture medium (90% McCoy’s 5A, 10% FBS, 1% L-glutamine, and 1% penicillin/streptomycin). SK-BR-3 cells viability was assessed by staining with acridine orange (AO) and propidium iodide (PI; ViaStain™ AO/PI Staining Solutions, Nexcelom) at three time points: immediately after enrichment, 48 h after enrichment, and 7 days after enrichment. AO-positive, PI-negative tumor cells were counted as viable cells, whereas AO-negative, PI-positive tumor cells were regarded as dead cells. The relative live cell ratio was calculated according to the following equation: % Relative live cell ratio = (number of viable SK-BR-3 cells/number of collected SK-BR-3 cells) ×100.

### 2.6 Clinical sample evaluation

In clinical sample validation experiments, whole-blood samples were obtained from 83 healthy subjects and 697 patients with malignant tumors at ShenZhen Cancer Hospital, Chinese Academy of Medical Sciences, and The First Affiliated Hospital of Guangdong Pharmaceutical University between December 2018 and October 2020. 125 patients were eliminated for the following reasons: sample hemolysis, coagulation, samples were out of storage time, unclear patient information and other factors. Another 20 patients from same institution were recruited for gene mutation analysis in CTCs. 5 patients were eliminated for hemolysis or coagulation. This research was conducted in accordance with the Declaration of Helsinki and was approved by the Clinical Research Ethics Committee of the National Cancer Center/National Clinical Research Center for Cancer/Cancer Hospital & Shenzhen Hospital, Chinese Academy of Medical Sciences, and Peking Union Medical College. All patients provided informed consent before blood sample collection, and all blood collection procedures were performed in accordance with the guidelines of venous blood specimen collection (WS/T 661-2020). Four milliliters of blood sample were collected in an acid-citrate-dextrose tube containing an anticoagulant and processed within 24 h to prevent clotting. The processed blood sample was transferred to a new sterile 15-mL centrifuge tube and centrifuged at 300× g for 10 min. The plasma was removed, and the remaining blood cells were resuspended in PBS. The resuspended sample was then slowly added to a Leucosep™ tube and subjected to gradient centrifugation at 1000× g for 10 min. The layer containing peripheral blood mononuclear cells (PBMC) was collected and resuspended again in PBS to a final volume of 2 mL.

### 2.7 Isolation of CTCs

CTC isolation were carried out in 4mL of whole blood using microfluidics platform (CTC100, Cellomics). The platform consists of a microfluidic inertial sorting chip containing a curved microchannel that depends on the balance of net lift force and dean force to sort cells according to cell size, shape and rigidity. PBMC (containing CTC cells) were obtained from the blood sample after density gradient centrifugation. Pumps was connected to the sample inlet of microfluidic chip and sterile tubes was connected to the CTC outlet and waste outlet. The PBMCs sample and PBS buffer were pumped into the microfluidic chip at the same time, screened by the chip, and the isolated CTCs were finally collected from the CTC outlet.

### 2.8 Immunofluorescence staining

The CTC collecting tube was centrifuged at 500× g for 10 min at room temperature. The supernatant was removed, and the cell pellet was gently resuspended in PBS buffer, attached by cytocentrifugation. The supernatant was removed, 200 µL of fixation buffer (4% paraformaldehyde solution) was added to the collected cells, and the buffer solution was incubated for 5 min. The cells were washed three times with PBS (5 min for each wash). The fixed cells were then permeabilized by 0.1% Triton X-100 dissolved in PBS buffer for 10 min and rinsed three times with PBS (5 min for each wash). The cells were then stained with PE-labelled anti-CD45 (1:100, Invitrogen), FITC-labelled anti-Pan-CK (1:200, Novus), and AF647-labelled anti-N-cadherin (1:100, Novus) by incubating overnight at 4°C. AF647-labelled anti-PD-L1 (1:100, Abcam), AF647-labelled anti-HER2 (1:100, Abcam), and AF647-labelled anti-vascular endothelial growth factor (VEGF; 1:100, Novus) antibodies were used instead of N-cadherin antibody for PD-L1, HER2, and VEGF assessment, respectively. After incubation, the cells were rinsed with PBS buffer three times and stained with DAPI for another 5 min. Finally, the collected CTCs were observed under a fluorescence microscope (Olympus IX73). The antibody used in this study was tested using positive and negative cell line controls, respectively. The performance of these antibodies is shown in **Table 1**.

**Table 1:** The performance of different antibodies tested by cell line controls.

Antibody	Positive control (Cell line)	Negative control (Cell line)	Sensitivity (%)	Specificity (%)
Pan CK	SK-BR-3	WBC	100	100
N-Cadherin	Hela	T47D	100	100
CD45	WBC	Hela	98	99
PD-L1	H226	MCF-7	96	98

### 2.9 Downstream gene analysis of isolated CTCs by Real-time quantitative polymerase chain reaction (qPCR)

The stained cells were transferred to a single-cell picking system. Using the microscope, CTCs were identified based on the criterion of DAPI+/CD45−/PanCK+/N-cadherin− for epithelial CTCs, DAPI+/CD45−/PanCK−/N-cadherin+ for mesenchymal CTCs, and DAPI+/CD45−/PanCK+/N-cadherin+ for E\M mixed-type CTCs. All the CTCs were then picked gently using a microneedle, transferred to the bottom of a polymerase chain reaction (PCR) tube, and centrifuged at 1500× g for 10 s. Next, 10 µL of protease lysis buffer (15 µL of proteinase K [20 mg/mL], 30 µL of 10× PCR buffer, and 30 µL of 25 mM MgCl_2_) was added to the PCR tube. CTCs were then digested at 56°C for 20 min and inactivated at 95°C for 20 min. Subsequently, 10 µL of 2× PCR reaction solution containing the corresponding primers was added to the PCR tube. The EGFR 19del primers were as follows: multiple forward primers: 5’-CCCGTCGCTATCAAAA-3’, 5’-CCCGTCGCTATCAAGAC-3’, 5’-CCCGTCGCTATCAAGGTT-3’, 5’-CCGTCGCTATCAAGGAGC-3’, 5’-CCCGTCGCTATCAAGGATC-3’, 5’-CCGTCGCTATCAAGGAAGC-3’, 5’-CCGTCGCTATCAAGGAACC-3’, 5’-CCGTCGCTATCAAGGAATC-3’; reverse primer: 5’-CCACACAGCAAAGCAGAAACTCA-3’. For internal control, we used the following primer: forward primer: 5’-GTTTGCCAAGGCACGAGTAAC-3’, and reverse primer: 5’-AAGGACCACCTCACAGTTATT-GAAC-3’. The KRAS G12D primers were as follows: forward primer: 5’-TGTGGTAGTTGGAGCTGA-3’; reverse primer: 5’-CCACACAGCAAAGCAGAAACTCA-3’. For internal control, we used the following primer: forward primer: 5’-GGAAATAAATGTGATTTGCCTTC-3’, and reverse primer: 5’-CCTGTCTTGTCTTTGCT-GATGTTTC-3’. The BRAF V600E primers were as follows: forward primer: 5’-TAGGTGATTTTGGTCTAGCTACAGA-3’, reverse primer: 5’-AGCCTCAATTCTTACCAT-CCAC-3’. For internal control, we used the following primer: forward primer: 5’-CTACCTTCATCTCTTTCAGTT-TTTC-3’, and reverse primer: 5’-GTTTGTTGGGCAGGAAGACTCTAAC-3’. The target gene was then measured using the QuantStudio™ Real-Time PCR system (Themo Fisher Scientific, USA), The amplification conditions were as follows: 95°C for 5 min, 95°C for 15 s/60°C for 60 s, and 40 cycles. After amplification, cycle threshold values were analyzed and interpreted accordingly.

### 2.10 Statistical analysis

Statistical analysis was performed using GraphPad Prism software, version 8.0. Two-tailed Student t-test was used for statistical comparison or one-way ANOVA test between groups. The sensitivity and specificity of CTC counts for classifying cancer patients versus healthy subjects were assessed using receiver operating characteristic (ROC) curve analysis. The area under the ROC curve (AUC) was calculated. The optimal cutoff points for CTC counts were obtained using the Youden Index method. The sensitivity and specificity of the CTC PD-L1 test in differentiating tissue-based PD-L1-positive cancer patients from tissue-based PD-L1-negative cancer patients were assessed using the ROC curve analysis. The AUC was calculated. Pearson’s correlation analysis was also performed. Statistical significance was defined as P<0.05.

## 3 Results

### 3.1 Workflow of CTC isolation and downstream analysis

Workflow of the microfluidic-based system for CTC isolation and downstream analysis are shown in **Figure 1**. In this study, we designed a CTC isolation and enrichment platform, CTC100 platform. The platform is based on a microfluidics chip that can isolate and enrich CTCs within 15 min from a 4 mL blood sample. A clinical protocol based on this platform was also established: firstly, a 4 mL venous blood sample was drawn from cancer patients; secondly, the PBMC layer was collected after density gradient centrifugation; then CTCs were enriched from the PBMC layer using the microfluidics-based platform; and in the last, phenotype/genotype analysis of the enriched CTCs was carried out (**Figure 1**).

### 3.2 Design and optimization of inertial focusing microfluidic chip

**Figure 2:**
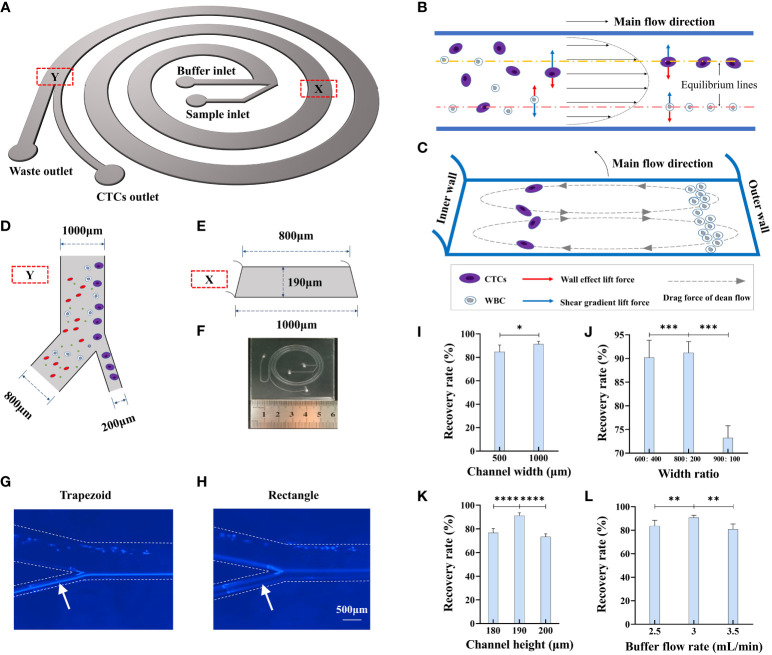
Design and working principle of CTC detection platform. **(A)** Schematic of trapezoidal cross-section spiral channel with two inlets and two outlets. **(B)** Cells in the channel migrate under influence of wall effect lift force and shear gradient lift force. **(C)** Cells in the channel are also affected by drag force from dean flow and migrate to sized dependent equilibrium points of the cross section. **(D)** Size of bifurcation in the channel. **(E)** Size parameter of the trapezoidal cross-section of the channel. **(F)** Size and configuration of trapezoidal microfluidic chip. **(G, H)** Flow track of Hela cell in the trapezoid and rectangle microfluidic chips. **(I)** The recovery rate of 15 μm microbeads in different width of channel, n=15. **(J)** The recovery rate of 15 μm microbeads in different width ratios of CTC outlet to waste outlet, n=15. **(K)** The recovery rate of 15 μm microbeads in different channel heights, n=15. **(L)** The recovery rate of 15 μm microbeads in different buffer flow rates, n=15. Data are presented as the mean ± SD, NS: not significant; *p < 0.05, **p < 0.01, ***p < 0.001, ****p < 0.0001 by one-way ANOVA test or two-tailed Student t-test, error bars indicate s.e.m.

Inertial focusing is a widely applied working principle in the field of microfluidics for processing fluids containing particles of different mechanical properties, including size, shape, and deformability (18). Currently available CTC separation platforms, some of which are based on inertial focusing, have drawbacks such as sample blockage, low CTC recovery rate, low recovery purity, and poor cell viability (13). To overcome these limitations, in this study, we designed a novel inertial focusing-based structure composed of a spiral microchannel with an isosceles trapezoidal cross-section (**Figures 2A–F**, SI1). Because all these forces are the functions of cell size, CTCs and normal blood cells migrate laterally to different equilibrium points of the cross-section. Such self-ordering provides an opportunity to separate tumor cells from blood cells according to cell size. By adding bifurcation, CTCs and WBCs can be separated from each other (**Figure 2D**). The flow track of HeLa cells in the different microfluidic chips was recorded using a microscope, which showed that the flow tracks of HeLa cells were more focused in the trapezoidal channel than in the rectangle channel (**Figures 2G, H**).

Several inertial focusing-based microfluidic chips are available for CTC enrichment (8, 19, 20), and most of them have a channel width ranging between 100 µm and 600 µm and sample processing time ranging from 8 min to 20 min. We compared the recovery rate and processing time for microfluidic chips with different channel widths, namely, 500 µm and 1000 µm, while the other parameters remained the same: channel height of 190 µm, width ratio of bifurcation of 1:4, and flow rate of 3 mL/min. As shown in **Figure 2I**, the corresponding recovery rates for the two microfluidic chips were 84.8 ± 3.4% and 91.2 ± 2.4%, while the processing time was nearly 1 h when the channel width was 500 µm, which was much longer than that for the microfluidic chip with a channel width of 1000 µm (processing time of less than 4 min). Thus, we set the channel width to 1000 µm because of the much shorter processing time and better recovery rate.

We next optimized the width ratio of bifurcation to effectively enrich the 15-µm microbeads,which can mimic CTC due to their similar biophysical characteristics in microfludics. We also used 9-µm microbeads to represent the WBCs as control. We designed various experiments to determine the optimal parameters associated with chip function, including the width of the microchannel, width ratio of bifurcation, channel height, and buffer flow rate. 1 mL of the 15-µm microbeads (10^5^/mL) and 1 mL of the 9-µm microbeads (10^6^/mL) was added into the sample inlet and fluid velocity was controlled through a microsyringe pump. We first compared microchannels of three different width ratios, namely, 400:600, 200:800, and 100:900, with recovery rates of 90.6±3.6%, 91.2±2.4%, and 73.2±2.5%, respectively (**Figure 2J**). The optimal output channel ratio was determined as 200:800. We then compared microchannels of three different channel heights, namely, 180 µm, 190 µm, and 200 µm, and the results showed that when the height was 190 µm, the 15-µm microbeads could be most effectively separated from the 9-µm microbeads (**Figure 2K**). The flow rate of the buffer in the microchannel is also an important factor for the separation efficiency of the microfluidic chip, as it decides the number of Dean cycles that the cells experience in the fixed-length channel. While the flow rate in the sample inlet was fixed at 600 µL/min, we compared microchannels of three different flow rates of buffer; the results showed that a flow rate of 3 mL/min had the best recovery rate for the 15-µm microbeads (**Figure 2L**). In the spiral microfluidic chip, smaller particles move from the outer side of the channel to the inner side and then return to the outer side again with a certain tendency of dispersion (i.e., they would have experienced a complete Dean cycle). As the flow continues, smaller particles slowly approach toward the outer wall, whereas larger particles gradually focus on the equilibrium position near the inner wall. This process could be affected by several parameters of the microchannel.

### 3.3 Separation and recovery of different tumor cell lines by trapezoid microfluidic chip

**Figure 3:**
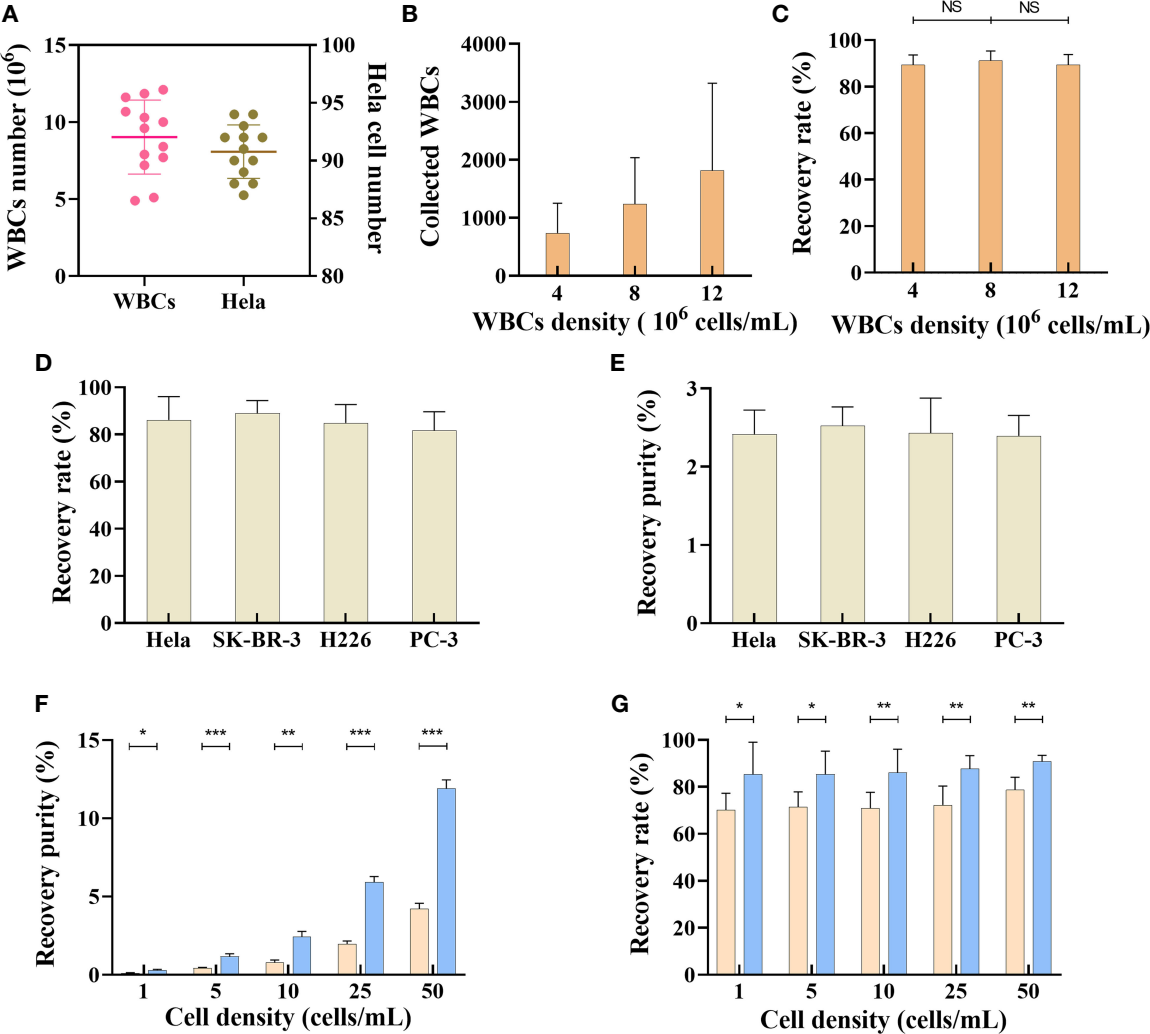
Separation of tumor cells by microfluidic chips. **(A)** WBCs and Hela cells number after density gradient centrifugation, n=13. Data are presented as the median with interquartile range. **(B)** The recovered number of WBCs from samples with different WBCs densities, n=15. **(C)** Recovery rate of SK-BR-3 tumor cell from samples with different WBCs densities, n=15. **(D)** Recovery rate of different tumor cell lines in trapezoid microfluidics chip, n=15. **(E)** Recovery purity of different tumor cell lines in trapezoid microfluidics chip, n=15. **(F)** Comparison of recovery purity of Hela cell in different tumor cell densities between trapezoid (blue bar) and rectangle (orange bar) microfluidics chips, n=15. **(G)** Comparison of recovery rate in different tumor cell densities between trapezoid (blue bar) and rectangle (orange bar) microfluidics chips, n=15. Data are presented as the mean ± SD, error bars indicate s.e.m. NS, not significant; *p < 0.05, **p < 0.01, ***p < 0.001 by two-tailed Student t-test.

This flow configuration has been feasible for CTC isolation, but the recovery rate has been severely compromised owing to the abundance of red blood cells in peripheral blood. In this study, we have addressed this problem by collecting the PBMC layer from the blood sample through density gradient centrifugation. The collected PBMC layer was then used for CTC isolation using the microfluidic chip, with a size threshold of 15 µm. As density gradient centrifugation may cause loss of tumor cells, we first evaluated the loss of tumor cells during this process. Briefly, 100 HeLa cells prestained with the CellTracker™ dye were added into a 4-mL (25 cells/mL) whole-blood sample for density gradient centrifugation. The stained HeLa cells were then counted, which revealed that the total number of HeLa cells in the PBMC layer was 91 ± 2 (**Figure 3A**), suggesting that density gradient centrifugation does not cause significant loss of tumor cells. Additionally, the WBC count in the PBMC layer ranged from approximately 4×10^6^ to 12×10^6^ cells/mL blood sample (**Figure 3A**). We next evaluated the effect of WBC counts on recovery rate of tumor cell lines. Nearly 100 prestained HeLa cells were added to 4 mL of PBS (25 cells/mL), with different densities of WBCs (4×10^6^ cells/mL, 8×10^6^ cells/mL, and 12×10^6^ cells/mL); the HeLa cells were then collected using the microfluidic chip. The results showed that under different WBC densities, the HeLa cell recovery rates were not significantly altered (**Figures 3B, C**).

We next evaluated the performance of this microfluidic platform for tumor cell lines of various cancer types. Briefly, samples (10 cells/mL) of different tumor cell lines were prepared by adding nearly 40 prestained HeLa cells, SK-BR-3 cells, H226 cells, and PC-3 cells into 4-mL whole-blood samples collected from healthy subjects. After subjecting the samples to density gradient centrifugation, the PBMC layer was collected for tumor cell isolation. As shown in **Figures 3D, E**, the recovery rates for different tumor cell lines were 86.2 ± 9.7%, 88.9 ± 5.3%, 84.9 ± 7.8%, and 81.6 ± 7.9%, respectively. The output purity of the collected tumor cells (HeLa cells, SK-BR-3 cells, H226 cells, and PC-3 cells) was 2.41 ± 0.31%, 2.52 ± 0.24%, 2.43 ± 0.44%, and 2.39 ± 0.26%, respectively. Various techniques, including fluorescence in situ hybridization, immunofluorescence staining (IF), and reverse transcription-PCR (RT-PCR)/qRT-PCR can be used to determine tumor cells (21–24). In this study, we established IF protocols to identify the collected tumor cells according to the following criteria: PanCK+, DAPI+, CD45- cells with a nucleus-to-cytoplasmic ratio of greater than 0.8.

CTC enrichment and separation require efficient removal of normal blood cells. Compared with a rectangle channel, a right-angled trapezoidal channel can change the shape of the velocity field and push the smaller WBCs closer to the outer wall, while the larger CTCs migrate to a position closer to the inner wall (19, 25–27). However, it is unclear whether an isosceles trapezoidal channel is better than a rectangle channel in terms of CTC enrichment. CellTracker™ dye prestained HeLa cells were spiked into whole-blood samples collected from healthy subjects to create different tumor cell densities (1-50 cells/mL blood) and isolated using the two kinds of chips. As the number of CTCs in cancer patients is usually between 0 and 200 cells/7.5 mL (28), we created the tumor cells density gradient from 0-50 cells/mL to compare the output purity and recovery rate between trapezoidal microchannel and rectangle microchannel. As shown in **Figure 3F**, the output purity of a microfluidic chip with a trapezoidal channel was 0.28±0.06% when the sample density was as low as 1 cell/mL (200-400 WBCs/mL, ∼4 log depletion of WBCs). The tumor cells purity increased when more HeLa cells were present in the samples. The output purity of the microfluidic chip with a trapezoidal channel was much higher than that with a rectangle channel, whose recovery purity was only 0.11±0.02% when the sample density was 1 cell/mL (**Figure 3F**). Our results also showed that the average overall recovery rate of tumor cells in a trapezoidal channel was 87.08±8.65%, independent of the CTC density in blood, whereas the recovery rate in a rectangle channel was lower than 80% under different HeLa cell densities (**Figure 3G**). These results suggest that the microfluidic chip with a trapezoidal channel had a significantly better performance than that with a rectangle channel.

### 3.4 Integrity and viability of tumor cells after separation

**Figure 4:**
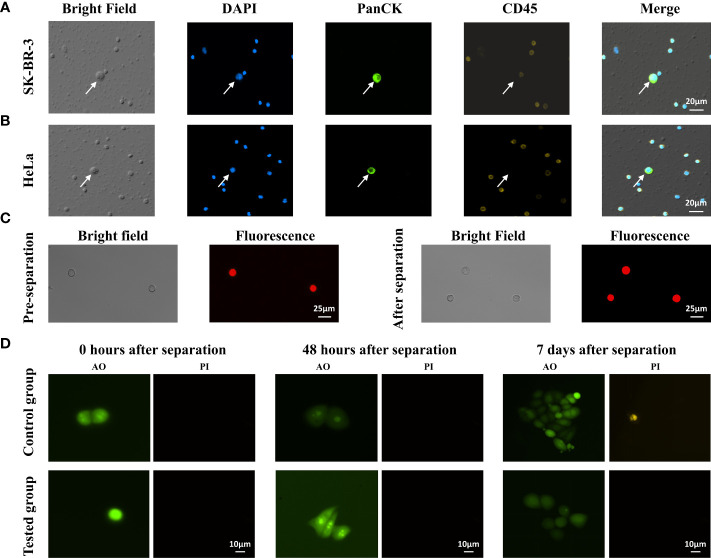
Recovery and identification of different tumor cell lines in trapezoid microfluidic chip. **(A)** Enriched SK-BR-3 cells and **(B)** Enriched Hela cells were characterized by immunostaining of DAPI, PanCK, CD45 antibodies, respectively. The arrows indicate the identified CTCs. **(C)** Observation of Hela cell morphologies before and after the separation by microfluid chip. **(D)** AO/PI staining of the collected SK-BR-3 cells at different times. Control group: SK-BR-3 cells without CTC100 separation; tested group: SK-BR-3 cells separated by CTC100.

After separation by trapezoid microfluidic chips, we performed immunofluorescence staining to identify different tumor cell lines. The collected SK-BR-3 cells and Hela cells were stained for DAPI, PanCK and CD45, and were observed and accurately identified under the microscope (Olympus IX73)(**Figures 4A, B**). Tumor cells showed PanCK+/CD45-, and WBCs showed PanCK-/CD45+. The size of tumor cell was much larger than WBCs.

Next, we assessed tumor cell integrity and viability after microfluidic separation. We found that no significant changes in morphology of Hela cells before and after separation (**Figure 4C**). The SK-BR-3 tumor cells were spiked into a 4-mL whole-blood sample collected from healthy subjects. The viability of the collected tumor cells at different time points after CTC enrichment was assessed by AO/PI staining. Immediately after enrichment, most of the collected tumor cells were AO positive and PI negative (**Figure 4D**), with relative live cell ratios of 95.05±3.54% in the control group and 90.55±4.79% in the test group (n=5). The relative live cell ratio decreased to approximately 41.2%-49.0% 48 h after CTC enrichment and maintained at a similar level until day 7 (n=5). For the control group, the relative live cell ratio decreased to 58.2%-61.5% from 48 h to 7 days (n=5). This indicated that microfluidics processing caused only a minor increase in cell death, and a large percentage of the collected tumor cells maintained their ability to divide and proliferate.

### 3.5 Clinical validation of the microfluidic CTC100 platform

Above results suggest that the microfluidics CTC100 platform could separate tumor cell lines with a higher recovery rate, output purity, integrity, and viability than other platforms (25, 29, 30). To validate the clinical utility of the protocol, we recruited 697 cancer patient and 83 healthy subjects for CTC enrichment and characterization. Among the cancer patients, 125 of them were eliminated due to the abnormal blood condition or other reasons (**Tables S3**). 4-mL whole-blood samples were obtained from each of the 572 cancer patients who differed by cancer types, stages, and treatments, and from 83 healthy subjects for CTC detection (**Table 2**; **Tables S4, S5**). For cancer patients, all blood samples were collected before starting anticancer therapy. The ability of the CTC100 platform to differentiate healthy subjects, and early, late stages of cancer patients based on CTC enumeration was evaluated, and the feasibility of detecting specific protein markers and target gene mutations in the enriched CTCs was also investigated.

**Table 2:** Representative characteristics of cancer patients and healthy subjects.

	N (percentage, %)
**Total patient numbers**	572
Age (years, median, range)	63 (27-94)
≥60	340
<60	232
Gender	
Male	279 (48.78)
Female	293 (51.22)
Group	
Non small cell lung cancer patient (NSCLC)	128 (22.38%)
Breast cancer patient (BC)	161 (28.15%)
Prostate cancer (PC)	166 (29.02%)
Pancreatic adenocarcinoma patient (PAAD)	53 (9.27%)
Esophageal carcinoma patient (EC)	59 (10.31%)
Hepatocellular cancer (HCC)	5 (0.87%)
**Total healthy subject number**	83
Age (years, median, range)	56 (28-77)
≥60	27
<60	56
Gender	
Male	42
Female	41

**Figure 5:**
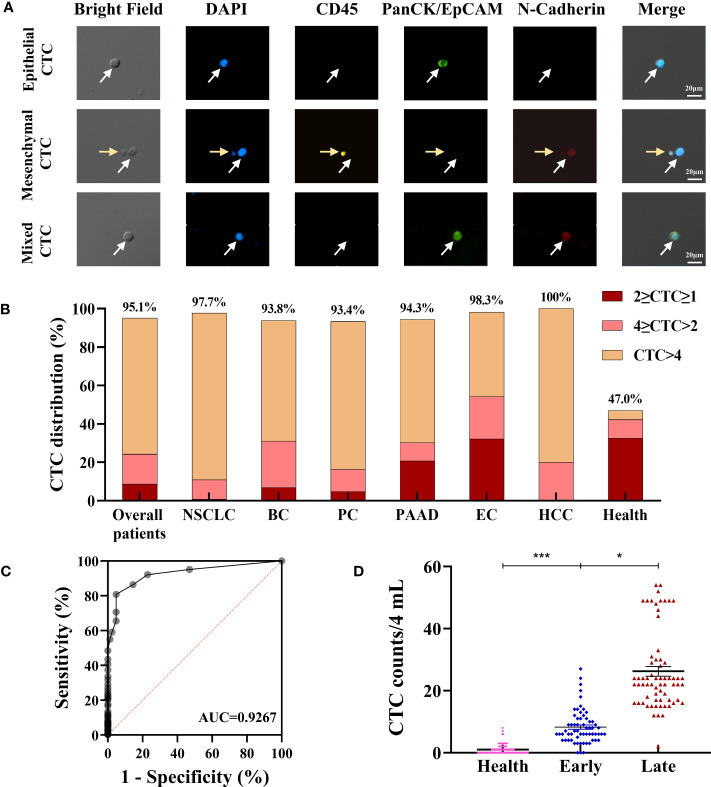
Evaluation of clinical utility of the CTC enrichment and characterization. **(A)** Phenotype feature of isolated CTCs: epithelial CTCs were defined as DAPI+/CD45-/PanCK+/N-Cadherin-; mesenchymal CTCs were defined as DAPI+/CD45-/PanCK-/N-Cadherin+; mixed type CTCs were defined as DAPI+/CD45-/PanCK+/N-Cadherin+. The white arrows indicate the CTCs, the yellow arrows indicate the WBCs. **(B)** CTC detection in cancer patients and healthy subjects. **(C)** ROC curve analysis of CTC counts in differentiating cancer patients from healthy subjects. **(D)** Difference of total CTC number in healthy subjects (n=83), early (n=62) and late (n=66) stage of NSCLC patients. Data are presented as the median with interquartile range; NS: not significant, *p < 0.05, **p < 0.01, ***p < 0.001 by two-tailed Student t-test; ROC: receive operating characteristic curve; AUC: area under the curve. Abbreviation: NSCLC, non-small cell lung cancer; BC, Breast cancer; PC, prostate cancer; PAAD, pancreatic adenocarcinoma; EC, esophageal cancer; HCC, Hepatocellular cancer.

EMT of CTCs is a complex process that occurs during tumor metastasis. It can be controlled by downregulating the the expression of the epithelial markers such as EpCAM or PanCK and upregulating the expression of the mesenchymal markers such as vimentin and N-cadherin. Based on the EMT process, CTCs can be subtyped into epithelial, mesenchymal, and mixed types. Determining the proportion of different CTC subtypes is clinically valuable, especially in the prognosis aspect (21, 31). In this study, we chose PanCK and N-cadherin as biomarkers for CTC subtyping based on previous studies (32, 33). As shown in **Figure 5A**, cells with DAPI+/CD45−/PanCK+/N-cadherin− were defined as epithelial CTCs; cells with DAPI+/CD45−/PanCK−/N-cadherin+ were defined as mesenchymal CTCs; cells with DAPI+/CD45−/PanCK+/N-cadherin+ were defined as E\M mixed-type CTCs; and cells with DAPI+/CD45+/PanCK−/N-cadherin− were defined as WBCs.

According to the above definition, CTCs were enriched, the results of 28 patients were 0 CTCs/4-mL blood samples, and CTCs were identified in the other 544 patients with CTC counts ranging from 1 to 520 CTCs/4-mL blood samples. The CTC detection rate (≥1 cells/4-mL blood) was as high as 95.10%, and most patients have more than 4 CTCs in their 4-mL blood samples (**Figure 5B**). The CTC positive rate was much higher than the detection rate reported in studies that used other platforms, which ranged from 17% in early-stage cancer patients to 75% in metastatic cancer patients (34–39). The ROC curve analysis of CTC counts for differentiating cancer patients from healthy subjects showed that the AUC was 0.9267. The threshold analysis by the Youden Index method suggested 4 CTCs/4 mL of the blood sample as the optimal cutoff value (**Figure 5C**), for cancer patients, CTC counts ≥4 suggests that ineffective intervention and/or higher possibility of malignancy metastasis, relapse and poor prognosis. Using this cutoff value, the sensitivity and specificity of the test were 80.77% and 95.18%, respectively. For healthy subjects, CTC is an auxiliary diagnostic index, comprehensive evaluation should be carried out in combination with other indicators, such as tumor markers and imaging methods. In previous studies, the EMT phenomenon has conferred CTCs with enhanced cell mobility, metastatic properties, and resistance to therapies (23). The number and percentage distribution of CTC subtypes in various cancer types assessed in this study are shown in **Table 3**, and the distribution characteristics will be further analyzed in the future for their value in tumor diagnosis and treatment.

**Table 3:** Number distribution of CTC subtypes in different cancer types.

Group	N (percentage, %)		
	Epithelial CTCs	Mesenchymal CTCs	Mixed CTCs
NSCLC	1059 (47.15)	769 (34.24)	418 (18.61)
BC	1115 (44.00)	947 (37.37)	472 (18.63)
PC	1317 (44.11)	1060 (35.50)	609 (20.40)
PAAD	302 (42.36)	267 (37.45)	144 (20.20)
EC	139 (39.83)	112 (32.09)	98 (28.08)
HCC	11 (40.74)	11 (40.74)	5 (18.52)

NSCLC, non-small cell lung cancer; BC, Breast cancer; PC, prostate cancer; PAAD, pancreatic adenocarcinoma; EC, esophageal cancer; HCC, Hepatocellular cancer.

We also compared the CTCs of 83 healthy subjects with those of the enrolled 128 non-small cell lung cancer (NSCLC) patients, which included 62 early-stage cancer patients and 66 late-stage cancer patients. The average CTC counts in healthy subjects, early-stage NSCLC (stages I and II) patients and late-stage NSCLC (stages III and IV) patients were 1.0 ± 1.6/4 mL, 8.3 ± 5.5/4 mL, and 26.3 ± 12.7/4 mL, respectively (**Figure 5D**). All the early-stage cancer patients had CTCs, and most of them (85.5%) had great than or equal to 4 CTCs. Interestingly, CTCs could also be identified in healthy subjects (**Figures 5B, D** **)**, and this may be due to the presence of aging nontumor cells, cells undergoing apoptosis, nonspecific immune reaction, or contaminated skin cells (28, 40, 41), but the number of CTCs in healthy subjects was significantly lower than that in NSCLC patients (P<0.05; **Figure 5D**). The average CTC counts in late-stage lung cancer patients was significantly higher than those in early-stage cancer patients and healthy individuals (**Figure 5D**). We also found that there was a significant difference in the distribution and average number of CTCs in BC patients with different stages (P<0.05, **Figure S1**), but not related with pathological molecular subtypes (**Figure S2**). These results indicate that the CTC counts captured by the CTC100 platform could serve as a biomarker for cancer diagnosis and monitoring.

### 3.6 Downstream analysis of enriched CTCs

Over the last decade, immune checkpoint inhibitors (ICIs) and targeted therapy have improved the progression-free and overall survival of cancer patients (42–44). Identifying specific biomarkers can aid in providing precise and individualized treatment to cancer patients. The expression of several proteins, such as PD-L1, HER2, and VEGF, has been recognized as a suitable predictive biomarker for monitoring tumor response to ICIs or targeted therapy (45–47). These biomarkers are traditionally assessed through the tissue-based immunohistochemistry method. However, tissue biopsy is invasive, and it is difficult to obtain the specimen; it may also cause high bleeding risk, which often prevents additional biopsies for immunohistological evaluation of these biomarkers (23). These disadvantages prevent dynamic monitoring and immune/targeted therapy selection in clinical practice (48).

Surface proteins of CTCs have been established as suitable biomarkers for ICIs or targeted therapies. A correlation between PD-L1 expression in CTCs and primary tumor development was reported (49), wherein PD-L1 expression in CTCs has been utilized as a biomarker for response to ICIs such as nivolumab and pembrolizumab. The predictive value of HER2 expression in CTCs was also evaluated in breast cancer, and CTC-based HER2-positive patients had higher survival under HER2-targeted therapy (50). VEGF could be a potential drug target (51). In this study, we demonstrated the ability of the platform to detect CTC membrane proteins (PD-L1, HER2, and VEGF) through IF assays and CTC genetic mutations (EGFR, KRAS, and BRAF) through real-time RT-PCR.

**Figure 6:**
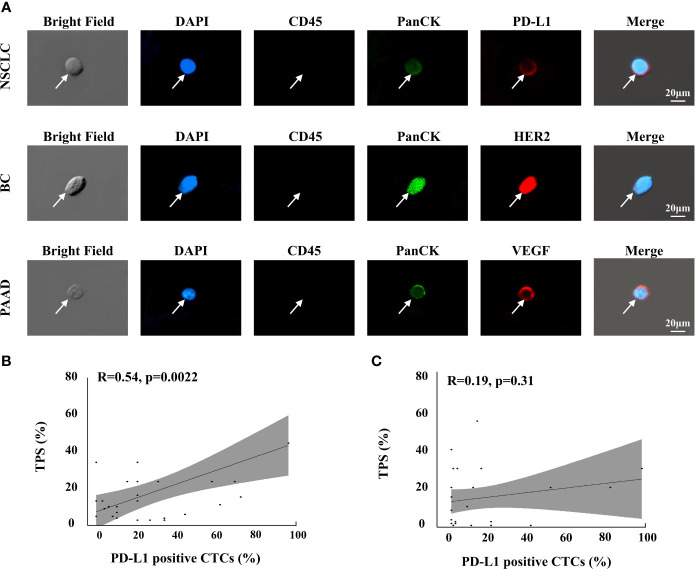
Protein analysis of isolated CTCs in cancer patients. **(A)** Phenotype feature of PD-L1, HER2, VEGF positive epithelial CTCs in NSCLC, BC and PAAD patients, respectively. **(B)** Correlations of the PD-L1 positive percentage of CTCs with the TPS of pathological PD-L1 testing in the group that the detection interval was shorter than 6 months, n=30 **(B)** and longer than 6 months, n=32 **(C)**.

As demonstrated in the previous section, the CTC enrichment platform developed in this study can preserve the integrity of the CTCs during the isolation process. This ensures the feasibility of performing downstream analysis of CTCs that originate from the tumor tissue. In this study, we successfully detected three clinically valuable protein biomarkers on CTCs, namely, PD-L1, HER2, and VEGF, from patients with NSCLC, breast cancer, and pancreatic adenocarcinoma (PAAD) (**Figure 6A**).

PD-L1 status was further assessed in CTCs from 62 late-stage NSCLC patients to compare with their tissue-based PD-L1 assessment. Antitumor therapies could change PD-L1 expression in cancer cells (52, 53). In this study, as the pathological PD-L1 tests were performed a few months ago and most patients received antitumor therapies during this period, we divided the NSCLC patients into two groups based on whether the detection interval between the tissue-based PD-L1 test and the CTC-based PD-L1 analysis was less than 6 months (n=30) or greater than 6 months (n=32). The correlation between the PD-L1-positive percentage of CTCs, which is calculated by the number of PD-L1-positive CTCs/number of total CTCs, and the tumor proportion score (TPS) of pathological PD-L1 results was assessed using Pearson’s correlation analysis. In the group where the detection interval was shorter than 6 months, the PD-L1-positive percentage of CTCs was associated with TPS (r=0.54, P=0.0022; **Figure 6B**), which suggested that the CTC-based PD-L1 analysis could be an alternative method for PD-L1 assessment. However, no such association was noted when the detection interval was longer than 6 months (r =0.19, P =0.31; **Figure 6C**); these findings may reflect the fact that long-term antitumor therapy had significantly changed the PD-L1 status of cancer cells, suggesting that CTC-based PD-L1 testing may be a choice when immunotherapy was considered while the latest pathological results were longer than 6 months ago.

Understanding the molecular basis and oncogenic drivers of cancer, such as EGFR, KRAS, and BRAF mutations, is crucial to providing targeted therapies for cancer patients (54) EGFR mutations are found in 10%-20% of lung adenocarcinoma cases (55) and are predictive of NSCLC response to erlotinib (56), a target drug that inhibits the tyrosine kinase activity of EGFR. KRAS and BRAF mutations are other two mutations widely assessed in various cancer types, including pancreatic cancer, colon cancer, and melanoma. Assessment of KRAS status is mandatory in patients with late-stage colorectal cancer (CRC) before initiating targeted therapy (57). BRAF mutation is present in up to 8% of cancer cases (58); specific BRAF inhibitors such as dabrafenib and vemurafenib were approved for use in metastatic colon cancer and melanoma (59). However, the decision to start targeted cancer therapies relies on the analysis of specific gene mutations, which is difficult to detect in metastatic or relapse cases years after the primary cancer diagnosis and surgical resection (60). CTCs provide a valuable source for studying whole-genome characterization in cancer, as they originate from both primary and metastatic tumors, and genetic analysis of CTCs for the presence or absence of key mutations may provide important clinical information over the treatment course to guide clinicians on when to stop or change the treatment plan (61–63). However, most of the currently available CTC capture approaches may not be very suitable for downstream genetic analysis owing to the difficulty in detaching the CTCs from the filter, morphological deformation of the CTCs, and very low rate of CTC purification (21, 64–67). The CTC enrichment platform developed in this study has the ability to enrich intact viable CTCs as unfixed cells in solution without the requirement of complex high-resolution imaging techniques or the use of expensive antibodies, which allows for the genetic analysis of CTCs using the conventional qPCR assay.

**Figure 7:**
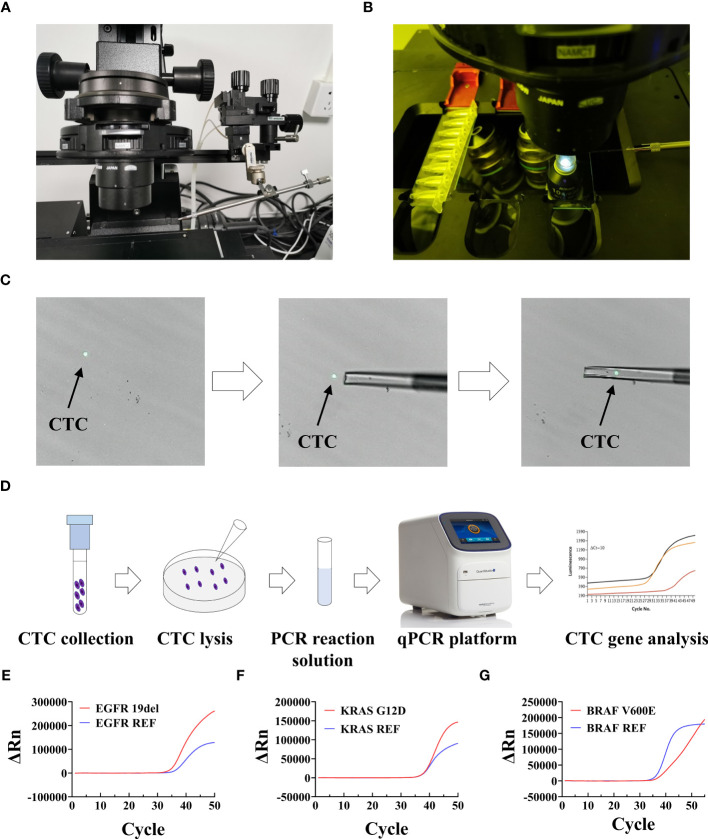
Setup and workflow of CTC picking and gene mutation analysis. **(A, B)** Setup of the single-cell picking system. **(C)** An illustration of the CTC picking process. **(D)** Workflow of fluorescence PCR for CTC genetic test. **(E–G)** Representative qPCR curves of analyzed mutations in collected CTCs. ΔRn is the magnitude of the normalized fluorescence signal generated by the reporter at each cycle during the PCR amplification.

After sorting and identification by CTC100, CTCs can be picked out by the single-cell picking system and then subjected to downstream single-cell analysis, such as gene mutation detected by qPCR assay, etc (**Figures 7A–D**). We first used this workflow on tumor cell lines, including H1650, PANC-1, and HT-29, to assess whether the most common genetic mutations in EGFR (19del), KRAS (G12D), and BRAF (V600E) (14, 58, 68). could be detected. Most of the cancer patients have more than four tumor cells, and approximately four tumor cells were spiked into a 4-mL healthy blood sample (1 cell/mL blood) for cell isolation. All the enriched tumor cells were then collected using the single-cell picking system, after which they were transferred into a PCR tube and tested using qPCR; the representative qPCR curves are shown in **Figures 7E–G**. The results showed that genetic mutations in EGFR, KRAS, and BRAF could be successfully detected (**Table 4**) in the collected tumor cells even when there was only one tumor cell (sample numbers 4 and 15). The CTC counts and genetic mutations carried by CTCs were then assessed in cancer patients who were confirmed to have the mutations based on tissue testing, including five EGFR 19del mutation-positive NSCLC samples, five KRAS G12D mutation-positive pancreatic adenocarcinoma patients, and five BRAF V600E mutation-positive CRC patients (**Tables S6**). As shown in **Table 5**, the genetic mutations were reliably detected in most of the patients. One exception is the CTC sample collected from a PAAD sample (sample number 6), which had a negative result, possibly because of tumor heterogeneity. These results demonstrated the great prospects of CTC genotyping, which may provide an alternative option for genetic testing and cancer monitoring in patients with advanced malignancy.

**Table 4:** Gene mutation in CTCs from cancer cell lines.

Test number	Cell line	Gene mutation	Amino Acid change	CTC counts (/4mL)	Result
1	H1650	EGFR	19del	5	Positive
2	H1650	EGFR	19del	2	Positive
3	H1650	EGFR	19del	3	Positive
4	H1650	EGFR	19del	1	Positive
5	H1650	EGFR	19del	4	Positive
6	PANC-1	KRAS	G12D	3	Positive
7	PANC-1	KRAS	G12D	4	Positive
8	PANC-1	KRAS	G12D	2	Positive
9	PANC-1	KRAS	G12D	6	Positive
10	PANC-1	KRAS	G12D	3	Positive
11	HT-29	BRAF	V600E	6	Positive
12	HT-29	BRAF	V600E	2	Positive
13	HT-29	BRAF	V600E	5	Positive
14	HT-29	BRAF	V600E	4	Positive
15	HT-29	BRAF	V600E	1	Positive

**Table 5:** Gene mutations in CTCs from cancer patients.

Sample number	Diagnosis	Stage	Pathological mutation	CTC counts (/4mL)	Tested mutation in CTCs	Result
1	NSCLC	IV	EGFR 19del	11	EGFR 19del	Positive
2	NSCLC	IV	EGFR 19del	40	EGFR 19del	Positive
3	NSCLC	IV	EGFR 19del	7	EGFR 19del	Positive
4	NSCLC	III	EGFR 19del	3	EGFR 19del	Positive
5	NSCLC	IV	EGFR 19del	14	EGFR 19del	Positive
6	PAAD	IV	KRAS G12D	3	KRAS G12D	Negative
7	PAAD	IV	KRAS G12D	5	KRAS G12D	Positive
8	PAAD	III	KRAS G12D	25	KRAS G12D	Positive
9	PAAD	IV	KRAS G12D	6	KRAS G12D	Positive
10	PAAD	IV	KRAS G12D	10	KRAS G12D	Positive
11	CRC	IV	BRAF V600E	8	BRAF V600E	Positive
12	CRC	III	BRAF V600E	5	BRAF V600E	Positive
13	CRC	IV	BRAF V600E	10	BRAF V600E	Positive
14	CRC	IV	BRAF V600E	11	BRAF V600E	Positive
15	CRC	IV	BRAF V600E	6	BRAF V600E	Positive

## 4 Discussion

In this study, we developed a novel inertial focusing-based system for efficient enrichment and identification of CTCs. In previous studies, spiral microfluidic channels with different dimensions, number of turns, and cross-sectional shapes had been implemented for CTC separation (18, 19, 69–71). With regard to the shape of the cross-section, trapezoidal spiral channels are a better choice, as they can induce the Dean vortex cores to shift toward the outer wall and consequently achieve better separation efficiency. In this study, a novel microfluidic-based platform involving isosceles trapezoidal spiral channels was utilized for CTC enrichment. It can separate CTCs from other cells in 4-mL whole-blood samples within 4 min and enrich rare CTCs even at a concentration of as low as 1 cell/mL (**Figure 3G**). When comparing the separation performances between the microfluidic chips with trapezoidal or rectangle cross-sections, our platform had a higher recovery rate and a better ability to purify CTCs (**Figures 2G, H**, **3F, G**).

EMT is a reversible cellular program that occurs when CTCs are circulating in the bloodstream. During this process, CTCs progressively lose their epithelial characteristic and gradually acquire more mesenchymal characteristics, which increases the invasive ability and elevates the resistance to antitumor therapies (72). Given that CTCs express both epithelial and mesenchymal biomarkers at varying levels, higher sensitivity strategies to isolate and enumerate CTCs based on their biophysical properties have been developed to capture CTCs in patients with low CTC counts (73). In this study, our system did not rely on tumor cell biomarkers such as epithelial markers (EpCAM, cytokeratins) or mesenchymal markers (vimentin, N-cadherin, E-cadherin). It could isolate all subtypes of CTCs, including epithelial, mesenchymal, and mixed types (**Figure 5A**). Given that this system is independent of tumor-associated biomarkers, the isolated CTCs are more representative of the original tumor, which is vital to the clinical application of liquid biopsy. As shown in **Figure 5A**, cells with DAPI+/CD45−/PanCK+/N-cadherin− were defined as epithelial CTCs; cells with DAPI+/CD45−/PanCK−/N-cadherin+ were defined as mesenchymal CTCs; cells with DAPI+/CD45−/PanCK+/N-cadherin+ were defined as E\M mixed-type CTCs; and cells with DAPI+/CD45+/PanCK−/N-cadherin− were defined as WBCs. As we know, CTCs expressing epithelial markers (EPCAM, cytokeratin (CK)) and lacking CD45 (a leukocyte marker) have been associated with poor outcome in many cancer types. However, recent study have shown that CK+/CD45+ (dual-positive) circulating cells are associated with prognosis in patients with advanced breast cancer, it is also a topic worth studying (74).

In this study, we also developed a CTC characterization protocol and explored its clinical utility in clinical samples. CTCs were enriched in most of the patient samples, with a 95.10% detection rate, independent of the clinical background, such as cancer type, stage, and treatment, which is much higher than those reported in other studies (75–78). When comparing with healthy subjects, CTC counts were significantly higher in cancer patients, and the AUC-ROC for CTC counts in discriminating between cancer patients and healthy subjects was 0.9267 (**Figure 5C**). At the cutoff point of 4 for cancer patients (CTC counts ≥4) or healthy subjects (CTC counts <4), sensitivity and specificity were 80.77% and 95.18%, respectively.

The correlations between CTC counts and subgroups with different clinicopathological features were investigated. Our results showed that most of the cancer samples have more than four CTCs. Thus, CTC counts could be used as a good indicator for differentiating healthy subjects from patients at different cancer stages (**Figure 5D**). The number of CTCs in healthy subjects were significantly lower than that in cancer patients (P<0.001; **Figure 5D**). Within the lung cancer patient group, a higher number of CTCs was associated with advanced tumor stages (P<0.05, **Figure 5D**). Patients with late-stage cancer showed a higher CTC counts and the consequent poor prognosis, which means that the CTC counts may be indicative of the prognosis of cancer patients. One key advantage of using CTCs is that it offers an opportunity to analyze tumor cells at the protein and gene levels, which could provide a more effective means to predict tumor response and treatment resistance. The major challenging issue in the accurate downstream molecular analysis of CTCs is the simultaneous enrichment and maintenance of the biological characteristics of CTCs. Another crucial advantage of our system is that the tumor cells are maintained in suspension or in flowing form throughout the isolation process, which preserves the integrity and viability of the isolated CTCs (**Figures 4C, D**). In this study, protein expression (PD-L1, HER2, and VEGF) on the cell surface and the genetic mutations (EGFR, KRAS, BRAF) within the CTCs were both successfully determined on the isolated CTCs, and the results showed good consistency with those obtained in tissue-based testing (**Figure 6**; **Table 5**).

Since CTCs were first discovered in 1869 (79), methods based on immunomagnetic capture were predominantly used, but clinical trials conducted in recent years have revealed the poor capture efficiency of these methods, which is less than 40% (79, 80). Over the past decades, microfluidic technologies based on various biological and physical principles have demonstrated great potential for CTC enrichment, especially in subsequent cellular and molecular assays (81). To date, many microfluidic-based methods are available for CTC enrichment. In this study, we compared our platform with several microfluidic-based CTC isolation platforms currently available on the market (**Table S2**) **(** 71–87). Most of the platforms that capture CTCs rely on immunocapture when CTCs flow at a low speed through the microchannel, wherein the throughput is moderate during a processing time of 60-300 min. We developed a novel CTC isolation platform that can enrich CTCs within 15 min as well as showing a higher capture efficiency (87.1%). The CTC detection rate with our platform is 95.10%, independent of the cancer stage and cancer type, which is higher than that obtained with most of the currently available platforms, implying its greater sensitivity for clinical utility. Finally, this platform can also effectively deplete leukocytes for subsequent CTC molecular analyses, including gene analysis, protein analysis, and CTC culturing.

In summary, our study demonstrates that our platform provides a reliable and efficient method for both sensitive CTC enumeration and downstream molecular analysis, allowing for rapid and cost-effective monitoring of tumor cell protein and gene features. Our results suggest that this platform has great potential for clinical applications and can aid in making informed decisions for patient care.

## 5 Conclusions

We have developed a microfluidic-based platform for CTC enrichment from whole-blood samples. Clinical validation experiments showed that the microfluidics platform could detect CTCs from 572 tumor patients, with a detection rate of 95.10%. ROC curve analysis confirmed the diagnostic value of this platform. The CTC counts can be used as an effective tool to compare and differentiate healthy subjects from patients with various lung diseases and cancer. Another crucial advantage of this system is that the integrity and viability of the CTCs are maintained with high purity. This enables direct downstream analysis of CTCs, including the detection of protein phenotypes and genetic mutations. In this study, we demonstrated the ability of the platform to detect CTC membrane proteins (PD-L1, HER2, and VEGF) through IF assays and CTC genetic mutations (EGFR, KRAS, and BRAF) through real-time RT-PCR.

These findings suggest that CTC-based molecular analysis may be a reliable approach for guiding precise oncotherapy, especially when the tumor tissue is clinically unavailable. We envision that this novel CTC capture platform could be a promising technique in the future to address the unmet clinical needs for CTC enrichment and personalized antitumor therapy.

## Data availability statement

The datasets generated and/or analyzed during the current study are not publicly available due to the privacy of patients but are available from the corresponding author on reasonable request. Requests to access these datasets should be directed to academic@cellomicsbio.com.

## Ethics statement

This research was conducted in accordance with the Declaration of Helsinki and was approved by the Clinical Research Ethics Committee of the National Cancer Center/ National Clinical Research Center for Cancer/Cancer Hospital & Shenzhen Hospital, Chinese Academy of Medical Sciences, and Peking Union Medical College. All patients provided informed consent before blood sample collection, and all blood collection procedures were performed in accordance with the guidelines of venous blood specimen collection (WS/T 661-2020). Informed consent was obtained from all subjects involved in the study.

## Author contributions

TX, XG, and ZY conceived the project, designed and supervised the experiments. SC, YD, and ZW2 performed the experiments and analyzed data, prepared figures, and wrote the manuscript. SC and CH conducted the major experiment of chip optimization and cell line experiments. YD and LD conduct the major experiment of clinical validation. ZW2, CW, and XY perform the major experiment of downstream analysis of the enriched CTC. WC and ZW1 helped analyze data, and prepared figures. LW and KM contributed to data acquisition, JZ and RH helped in designing and optimized the chip. HZ and WZ helped in cell viability experiment. YH, ZL, and TQ provided technical support for all the experiments. All authors contributed to the article and approved the submitted version.
