# Supplementary material for: Development and clinical validation of a microfluidic-based platform for CTC enrichment and downstream molecular analysis
Source: Front Oncol. 2023 Oct 2;13:1238332. doi: 10.3389/fonc.2023.1238332 (PMC10578963; doi:10.3389/fonc.2023.1238332)
Supplement: Supplementary file 1 [file DataSheet_1.docx]

Supplementary Material

Development and clinical validation of a microfluidics-based platform for CTC enrichment and downstream molecular analysis

Songhua Cai^a,1^, Youjun Deng^a,1^, Zhe Wang^b,1^, Junyu Zhu^c^, Chujian Huang^a^, Longde Du^a^, Chunguang Wang^a^, Xiangyang Yu^a^, Wenyi Liu^a^, Chenglin Yang^a^, Zhe Wang^a^, Lixu Wang^a^, Kai Ma^a^, Rui Huang^d^, Xiaoyu Zhou^e^, Heng Zou^e^ , Wenchong Zhang^e^ , Yan Huang^e^ , Zhi Li^e^ , Tiaoping Qin^e^, Tao Xu ^e,^*, Xiaotong Guo ^a,^*, Zhentao Yu^a,^*

*** Correspondence:**

Tao Xu [(academic@cellomicsmed.com);](mailto:(academic@cellomicsmed.com);) Xiaotong Guo [(guoxiaotong@chcamssz.ac.cn);](mailto:(guoxiaotong@chcamssz.ac.cn);) Zhentao Yu (yuzhentao@chcamssz.ac.cn).

# Supplementary Information

**Supplementary Information 1. Design and working principle of CTCs detection platform.**

The curvature radius of the chip is 15 mm, and the main channel width is 1 mm. The structure has two inlets (for sample and buffer) and two outlets (for CTC collection and waste output; Figure 2a,d-f). Initially, the sample and the buffer solution are pumped into the microchannel through the sample inlet and the buffer inlet, respectively. When the cells of different sizes flow into the microchannel, they experience inertial lift forces (FL) from the nature of laminar velocity, which is the net of two forces, namely, shear-induced lift force (FIL) and wall-induced lift force (FWL; Figure 2b), as defined in Eq. (1):

$$F_{L}=\frac{\rho U_{m}^{2}a_{p}^{4}}{D_{h}^{2}}C_{L}$$

where ρ is the fluid density, Um is the maximum velocity, Dh is the hydraulic diameter of the channel, CL is the lift coefficient, and ap is the particle diameter. The spiral geometry of the microchannel could produce two eddy currents (Dean flow) with opposite rotation directions, where both currents are vertical to the main flow direction (Figure 2c). This orientation produces a third drag force (Figure 2c) from the Dean flow on the cells; consequently, when the cells are flowing along the main flow direction, they will also migrate to a specific equilibrium position determined by the ratio of the three forces (Rf; Figure 2b, 2c), as defined in Eq. (2):

$$Rf=2Ra_{p}^{2}/D_{h}^{3}$$

where R is the hydraulic diameter of the curvature, Dh is the hydraulic diameter of the channel, and ap is the particle diameter.

# Supplementary Figures


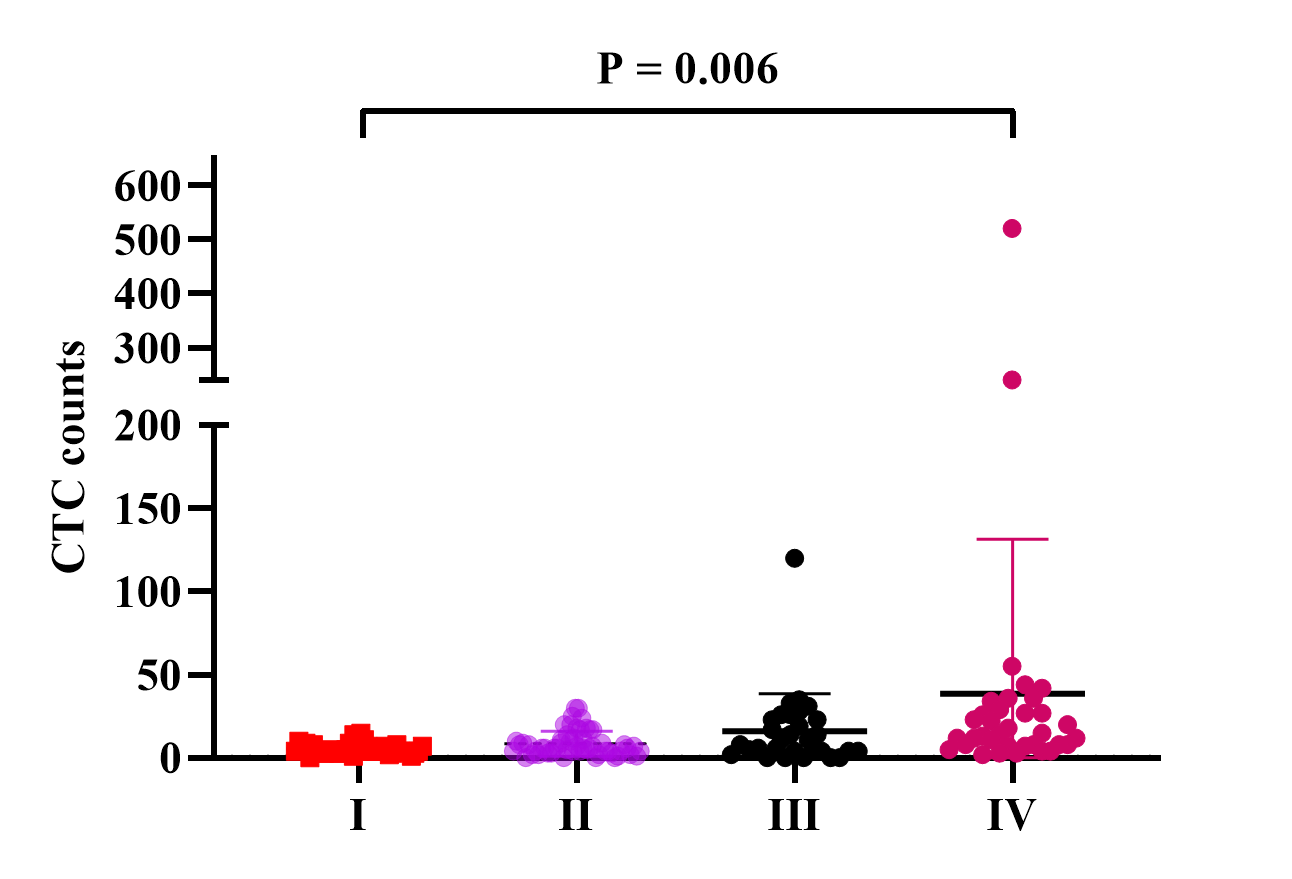


**Supplementary Figure 1. Distribution of CTC number in different stages of breast cancer.**

The number of CTCs are related to breast cancer stages, P=0.006 (one-way ANOVA

analysis).

**
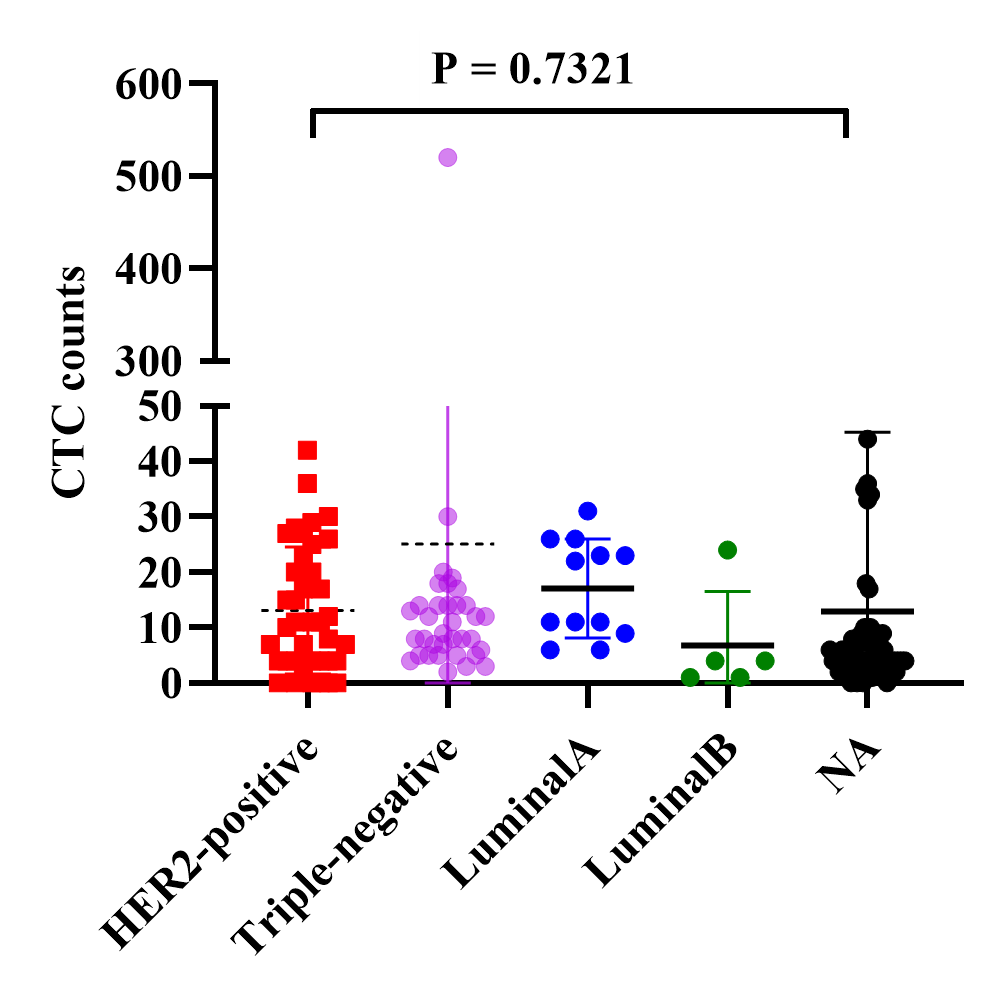
**

**Supplementary Figure 2. Distribution of CTC number in different molecular subtypes of breast cancer.** The number of CTCs are related to molecular subtypes of breast cancer, P=0.7321 (one-way ANOVA analysis).

# Supplementary Tables

**Supplementary Table 1.**  **Comparison of currently available CTC isolation techniques**

| **Method** | **Type** | **Advantage** | **Disadvantage** |
| --- | --- | --- | --- |
| Microﬁltration | - Label free | - Rapid processing - High eﬃciency | - Low purity - Membrane clogging - Diﬀerent size of CTCs - Diﬃcult to detach CTC from the ﬁlter for downstream analysis |
| Inertial Focusing | - Label free | - Precise - High throughput - Simple structure | - Complicated principle - Morphological deformation of captured cells |
| Dielectrophoresis | - Label free | - Single-cell isolation - High cell viability - High efficiency | - Slow processing - Low purity - Cell electrical properties can be affected |
| Direct Imaging | - Label free | - High-resolution fluorescence imaging | - Time-consuming |
| Magnetic Nanoparticles | - Label dependent | - Multiplexed processing - High capture and enrichment eﬃciency | - Expensive - Low cell viability - Dependence on expressed proteins - Difficulty for downstream analysis |
| Microfluidic and immunomagnetic based capturing | - Label dependent | - high enrichment eﬃciency - High purity | - Expensive - Moderate sensitivity - Slow ﬂow rate - Diﬃcult to detach CTC from chip for downstream analysis |

**Supplementary Table 2.** **List of microfluidic-based CTC enrichment methods with their characteristics.**

| **Platform** | **Working principle** | **Processing time (minutes)** | **Detection rate（%）** | **Capture efficiency（%）** | **Leukocyte depletion rate (fold)** | **Downstream molecular analysis** | **Ref.** |
| --- | --- | --- | --- | --- | --- | --- | --- |
| A | EpCAM antibody immobilized  microfluidic chip | 240 | 75-95 | 92 | NA | NA | [68, 69] |
| B | Size-based filtration in microfluidic chip | 30 | 94 | 85 | NA | Gene, Protein | [70, 72] |
| C | Antibody immobilized  microfluidic chip | 180 | 95.1 | 78-85 | 103 | Gene, Protein | [73-75] |
| D | Size-base Dean Flow Fractionation (DFF) (inertial focusing) | 70 | 79-94 | 80.4 | 10^2^ | Gene, Protein, Culture | [76-80] |
| E | Microscale  vortices and inertial focusing | 60-120 | 73-90 | 71 | 10^3^ | Gene, Protein, Culture | [81] |
| F | Size-based enrichment with either EpCAM-based positive enrichment or CD45 negative  depletion | 60-300 | 99 | 65 | 10^3^ | Gene, Protein | [82] |
| G | Antibody immobilized  microfluidic chip | 210 | 86.8 | 73 | NA | Protein | [83, 84] |
| This study | Size-base Dean Flow Fractionation (DFF) (inertial focusing) | 15 | 95.8 | 87.1 | 10^4^ | Gene, Protein, Culture |  |

**Supplementary Table 3. Clinical information of 125 patients who were excluded**

| Patient number | Sex | Age | Diagnosis | Date sample received | Sample state |
| --- | --- | --- | --- | --- | --- |
| 1 | M | 63 | NSCLC | 2018-7-26 | hemolysis |
| 2 | F | 59 | NSCLC | 2018-7-26 | hemolysis |
| 3 | M | 65 | PAAD | 2018-9-17 | hemolysis |
| 4 | F | 34 | BC | 2018-11-11 | Storage time exceeded |
| 5 | NA | NA | NSCLC | 2018-11-13 | nomal |
| 6 | F | 43 | NA | 2018-11-17 | nomal |
| 7 | NA | NA | BC | 2018-11-18 | nomal |
| 8 | M | 49 | NSCLC | 2018-11-25 | hemolysis |
| 9 | M | 76 | PAAD | 2018-12-14 | coagulation |
| 10 | M | 72 | NSCLC | 2019-1-15 | coagulation |
| 11 | M | 64 | NSCLC | 2019-1-17 | hemolysis |
| 12 | M | 62 | PAAD | 2019-3-1 | hemolysis and coagulation |
| 13 | NA | NA | PAAD | 2019-3-4 | nomal |
| 14 | M | 68 | PAAD | 2019-3-4 | hemolysis |
| 15 | NA | NA | NSCLC | 2019-4-11 | nomal |
| 16 | M | 54 | PAAD | 2019-4-22 | hemolysis |
| 17 | NA | NA | NSCLC | 2019-4-26 | nomal |
| 18 | M | 59 | NSCLC | 2019-4-29 | coagulation |
| 19 | M | 53 | PAAD | 2019-5-16 | coagulation |
| 20 | M | 71 | NSCLC | 2019-5-16 | Storage time exceeded |
| 21 | M | 63 | NSCLC | 2019-5-16 | coagulation |
| 22 | F | 84 | NA | 2019-5-16 | nomal |
| 23 | M | 47 | NSCLC | 2019-5-18 | Storage time exceeded |
| 24 | F | 62 | PAAD | 2019-5-19 | coagulation |
| 25 | NA | NA | NSCLC | 2019-5-21 | nomal |
| 26 | M | 57 | NSCLC | 2019-5-24 | hemolysis |
| 27 | F | 48 | PAAD | 2019-5-26 | coagulation |
| 28 | M | 71 | NSCLC | 2019-5-26 | hemolysis and coagulation |
| 29 | M | 66 | NSCLC | 2019-5-27 | coagulation |
| 30 | M | 65 | NSCLC | 2019-5-27 | hemolysis |
| 31 | F | 46 | PAAD | 2019-5-30 | coagulation |
| 32 | M | 51 | NSCLC | 2019-5-31 | coagulation |
| 33 | F | 55 | NSCLC | 2019-6-1 | hemolysis |
| 34 | NA | NA | NSCLC | 2019-6-5 | nomal |
| 35 | M | 58 | NSCLC | 2019-6-5 | coagulation |
| 36 | M | 71 | NSCLC | 2019-6-7 | hemolysis and coagulation |
| 37 | M | 59 | PAAD | 2019-6-10 | hemolysis |
| 38 | M | 59 | NSCLC | 2019-6-13 | hemolysis |
| 39 | F | 73 | NSCLC | 2019-6-15 | hemolysis and coagulation |
| 40 | M | 71 | NA | 2019-6-15 | nomal |
| 41 | NA | NA | NSCLC | 2019-6-15 | nomal |
| 42 | M | 65 | NSCLC | 2019-6-16 | coagulation |
| 43 | M | 70 | NSCLC | 2019-6-16 | coagulation |
| 44 | NA | NA | NSCLC | 2019-6-16 | nomal |
| 45 | M | 57 | NSCLC | 2019-6-16 | coagulation |
| 46 | M | 57 | NSCLC | 2019-6-16 | hemolysis |
| 47 | M | 59 | NSCLC | 2019-6-19 | hemolysis and coagulation |
| 48 | F | 49 | PAAD | 2019-6-24 | hemolysis and coagulation |
| 49 | M | 30 | NSCLC | 2019-7-7 | coagulation |
| 50 | M | 39 | NSCLC | 2019-7-7 | hemolysis and coagulation |
| 51 | M | 74 | NA | 2019-7-8 | nomal |
| 52 | M | 43 | NSCLC | 2019-7-8 | hemolysis |
| 53 | NA | NA | NSCLC | 2019-7-9 | nomal |
| 54 | M | 66 | NA | 2019-7-11 | nomal |
| 55 | M | 71 | NSCLC | 2019-7-12 | coagulation |
| 56 | M | 666 | NSCLC | 2019-7-23 | hemolysis |
| 57 | M | 71 | PAAD | 2019-8-6 | coagulation |
| 58 | F | 31 | PAAD | 2019-8-14 | coagulation |
| 59 | M | 51 | PAAD | 2019-8-31 | coagulation |
| 60 | M | 70 | NSCLC | 2019-9-26 | hemolysis and coagulation |
| 61 | M | 59 | NA | 2019-9-28 | nomal |
| 62 | M | 30 | NSCLC | 2019-10-10 | coagulation |
| 63 | M | 77 | NA | 2019-10-10 | nomal |
| 64 | F | 75 | NSCLC | 2019-10-12 | hemolysis |
| 65 | M | 76 | NA | 2019-10-13 | nomal |
| 66 | M | 59 | NSCLC | 2019-10-22 | hemolysis |
| 67 | M | 69 | NSCLC | 2019-10-27 | Storage time exceeded |
| 68 | M | 46 | PC | 2019-11-2 | hemolysis and coagulation |
| 69 | M | 58 | NSCLC | 2019-11-9 | Storage time exceeded |
| 70 | M | 70 | NSCLC | 2019-11-9 | Storage time exceeded |
| 71 | F | 37 | BC | 2019-11-9 | coagulation |
| 72 | M | 74 | NSCLC | 2019-11-10 | hemolysis |
| 73 | M | 57 | NA | 2019-11-10 | nomal |
| 74 | F | 49 | BC | 2019-11-10 | coagulation |
| 75 | M | 73 | NSCLC | 2019-11-11 | hemolysis |
| 76 | M | 85 | PC | 2019-11-12 | hemolysis |
| 77 | M | 76 | NSCLC | 2019-11-13 | Storage time exceeded |
| 78 | M | 66 | NSCLC | 2019-11-13 | coagulation |
| 79 | F | 56 | EC | 2019-11-16 | hemolysis |
| 80 | M | 68 | NSCLC | 2019-11-18 | hemolysis and coagulation |
| 81 | F | 38 | BC | 2019-11-30 | hemolysis |
| 82 | F | 40 | NSCLC | 2019-11-30 | Storage time exceeded |
| 83 | F | 50 | BC | 2019-12-5 | hemolysis |
| 84 | F | 60 | NSCLC | 2019-12-6 | coagulation |
| 85 | NA | NA | PC | 2019-12-8 | nomal |
| 86 | M | 51 | NSCLC | 2019-12-16 | hemolysis and coagulation |
| 87 | M | 12 | EC | 2020-1-18 | hemolysis |
| 88 | M | 54 | NSCLC | 2020-2-3 | hemolysis |
| 89 | M | 73 | PC | 2020-2-7 | hemolysis and coagulation |
| 90 | F | 52 | BC | 2020-2-8 | hemolysis |
| 91 | M | 51 | NSCLC | 2020-2-10 | coagulation |
| 92 | M | 30 | NA | 2020-2-14 | nomal |
| 93 | M | 35 | EC | 2020-3-11 | hemolysis |
| 94 | M | 46 | EC | 2020-3-16 | hemolysis |
| 95 | F | 44 | EC | 2020-3-16 | coagulation |
| 96 | F | 72 | PC | 2020-3-17 | hemolysis |
| 97 | M | 59 | PC | 2020-3-17 | coagulation |
| 98 | M | 59 | PC | 2020-3-17 | hemolysis |
| 99 | M | 72 | NSCLC | 2020-3-18 | Storage time exceeded |
| 100 | F | 50 | BC | 2020-3-20 | hemolysis and coagulation |
| 101 | M | 73 | NSCLC | 2020-3-22 | coagulation |
| 102 | M | 81 | PC | 2020-3-24 | hemolysis |
| 103 | M | 66 | PC | 2020-3-29 | hemolysis |
| 104 | M | 44 | EC | 2020-3-30 | coagulation |
| 105 | M | 73 | EC | 2020-3-30 | coagulation |
| 106 | M | 72 | EC | 2020-3-30 | coagulation |
| 107 | M | 66 | PC | 2020-4-6 | hemolysis |
| 108 | M | 68 | PC | 2020-4-6 | hemolysis |
| 109 | M | 38 | EC | 2020-4-6 | coagulation |
| 110 | M | 44 | EC | 2020-4-6 | coagulation |
| 111 | M | 57 | EC | 2020-4-6 | coagulation |
| 112 | M | 69 | PAAD | 2020-4-16 | coagulation |
| 113 | M | 67 | NSCLC | 2020-4-19 | coagulation |
| 114 | M | 34 | PAAD | 2020-4-20 | coagulation |
| 115 | M | 62 | PC | 2020-4-21 | hemolysis and coagulation |
| 116 | F | 59 | PAAD | 2020-4-21 | hemolysis and coagulation |
| 117 | F | 41 | BC | 2020-4-21 | hemolysis |
| 118 | M | 69 | NSCLC | 2020-4-23 | coagulation |
| 119 | M | 40 | EC | 2020-4-28 | coagulation |
| 120 | F | 65 | BC | 2020-5-10 | hemolysis |
| 121 | M | 35 | PAAD | 2020-5-11 | coagulation |
| 122 | M | 52 | NSCLC | 2020-5-19 | hemolysis and coagulation |
| 123 | F | 70 | NA | 2020-5-25 | nomal |
| 124 | M | 68 | PAAD | 2020-5-28 | coagulation |
| 125 | F | 59 | PAAD | 2020-5-31 | coagulation |

**Supplementary Table 4. Clinical information and CTCs results of patients**

| Patient number | Sex | Age | Diagnosis | Stage | Detection time | Total CTCs (/4mL) | Epithelial CTCs (/4mL) | Mesenchymal CTCs (/4mL) | Mixed CTCs (/4mL) |
| --- | --- | --- | --- | --- | --- | --- | --- | --- | --- |
| 1 | Male | 73 | NSCLC | I | 2019-07-03 | 3 | 2 | 1 | 0 |
| 2 | Male | 29 | NSCLC | I | 2019-07-04 | 0 | 0 | 0 | 0 |
| 3 | Male | 56 | NSCLC | I | 2019-07-04 | 4 | 1 | 2 | 1 |
| 4 | Female | 57 | NSCLC | I | 2019-07-04 | 27 | 12 | 10 | 5 |
| 5 | Male | 30 | NSCLC | I | 2019-07-04 | 13 | 6 | 2 | 5 |
| 6 | Male | 66 | NSCLC | I | 2019-07-04 | 3 | 2 | 1 | 0 |
| 7 | Male | 67 | NSCLC | I | 2019-07-05 | 6 | 1 | 4 | 1 |
| 8 | Male | 49 | NSCLC | I | 2019-07-05 | 3 | 2 | 0 | 1 |
| 9 | Male | 67 | NSCLC | I | 2019-07-05 | 7 | 4 | 1 | 2 |
| 10 | Male | 63 | NSCLC | I | 2019-07-09 | 4 | 2 | 1 | 1 |
| 11 | Female | 69 | NSCLC | I | 2019-07-09 | 20 | 12 | 5 | 3 |
| 12 | Female | 48 | NSCLC | I | 2019-07-10 | 13 | 6 | 4 | 3 |
| 13 | Male | 53 | NSCLC | I | 2019-07-11 | 8 | 5 | 2 | 1 |
| 14 | Female | 56 | NSCLC | I | 2019-07-11 | 6 | 3 | 3 | 0 |
| 15 | Female | 55 | NSCLC | I | 2019-07-16 | 8 | 4 | 3 | 1 |
| 16 | Female | 78 | NSCLC | I | 2019-07-17 | 6 | 2 | 2 | 2 |
| 17 | Male | 64 | NSCLC | I | 2019-07-17 | 11 | 5 | 3 | 3 |
| 18 | Male | 85 | NSCLC | I | 2019-07-18 | 0 | 0 | 0 | 0 |
| 19 | Male | 69 | NSCLC | I | 2019-07-18 | 4 | 2 | 1 | 1 |
| 20 | Male | 65 | NSCLC | I | 2019-07-19 | 22 | 10 | 7 | 5 |
| 21 | Female | 73 | NSCLC | I | 2019-07-19 | 9 | 5 | 3 | 1 |
| 22 | Female | 74 | NSCLC | I | 2019-07-19 | 8 | 3 | 4 | 1 |
| 23 | Male | 74 | NSCLC | I | 2019-07-19 | 6 | 2 | 3 | 1 |
| 24 | Male | 70 | NSCLC | I | 2019-07-23 | 14 | 3 | 7 | 4 |
| 25 | Male | 75 | NSCLC | I | 2019-07-23 | 6 | 2 | 3 | 1 |
| 26 | Female | 78 | NSCLC | I | 2019-07-24 | 9 | 5 | 2 | 2 |
| 27 | Male | 65 | NSCLC | I | 2019-07-24 | 15 | 6 | 6 | 3 |
| 28 | Male | 65 | NSCLC | I | 2019-07-24 | 4 | 2 | 1 | 1 |
| 29 | Male | 73 | NSCLC | I | 2019-07-25 | 6 | 3 | 1 | 2 |
| 30 | Female | 40 | NSCLC | I | 2019-07-25 | 12 | 6 | 3 | 3 |
| 31 | Male | 62 | NSCLC | II | 2019-07-25 | 4 | 2 | 2 | 0 |
| 32 | Male | 67 | NSCLC | II | 2019-07-26 | 24 | 9 | 8 | 7 |
| 33 | Female | 51 | NSCLC | II | 2019-07-26 | 11 | 5 | 3 | 3 |
| 34 | Male | 56 | NSCLC | II | 2019-07-26 | 6 | 3 | 1 | 2 |
| 35 | Female | 48 | NSCLC | II | 2019-07-31 | 6 | 2 | 3 | 1 |
| 36 | Male | 64 | NSCLC | II | 2019-07-31 | 7 | 2 | 3 | 2 |
| 37 | Female | 60 | NSCLC | II | 2019-07-31 | 6 | 5 | 1 | 0 |
| 38 | Female | 69 | NSCLC | II | 2019-07-31 | 14 | 7 | 3 | 4 |
| 39 | Female | 45 | NSCLC | II | 2019-07-31 | 3 | 3 | 0 | 0 |
| 40 | Male | 63 | NSCLC | II | 2019-08-01 | 4 | 3 | 1 | 0 |
| 41 | Male | 37 | NSCLC | II | 2019-08-01 | 14 | 6 | 6 | 2 |
| 42 | Male | 78 | NSCLC | II | 2019-08-05 | 9 | 6 | 2 | 1 |
| 43 | Male | 49 | NSCLC | II | 2019-08-05 | 3 | 2 | 1 | 0 |
| 44 | Male | 72 | NSCLC | II | 2019-08-05 | 6 | 3 | 3 | 0 |
| 45 | Male | 35 | NSCLC | II | 2019-08-06 | 18 | 9 | 8 | 1 |
| 46 | Female | 54 | NSCLC | II | 2019-08-06 | 6 | 2 | 2 | 2 |
| 47 | Female | 47 | NSCLC | II | 2019-08-06 | 9 | 3 | 2 | 4 |
| 48 | Male | 58 | NSCLC | II | 2019-08-07 | 5 | 3 | 2 | 0 |
| 49 | Male | 60 | NSCLC | II | 2019-08-08 | 4 | 2 | 2 | 0 |
| 50 | Female | 47 | NSCLC | II | 2019-08-12 | 0 | 0 | 0 | 0 |
| 51 | Male | 50 | NSCLC | II | 2019-08-12 | 11 | 5 | 4 | 2 |
| 52 | Male | 52 | NSCLC | II | 2019-08-12 | 3 | 2 | 1 | 0 |
| 53 | Female | 57 | NSCLC | II | 2019-08-14 | 6 | 2 | 2 | 2 |
| 54 | Female | 65 | NSCLC | II | 2019-08-21 | 10 | 5 | 3 | 2 |
| 55 | Female | 74 | NSCLC | II | 2019-08-28 | 6 | 2 | 2 | 2 |
| 56 | Male | 46 | NSCLC | II | 2019-09-04 | 12 | 5 | 5 | 2 |
| 57 | Male | 49 | NSCLC | II | 2019-09-09 | 9 | 3 | 5 | 1 |
| 58 | Female | 45 | NSCLC | II | 2019-09-09 | 10 | 5 | 3 | 2 |
| 59 | Female | 35 | NSCLC | II | 2019-09-11 | 8 | 4 | 3 | 1 |
| 60 | Female | 56 | NSCLC | II | 2019-09-12 | 9 | 5 | 2 | 2 |
| 61 | Male | 46 | NSCLC | II | 2019-09-19 | 7 | 3 | 3 | 1 |
| 62 | Female | 63 | NSCLC | II | 2019-09-19 | 6 | 3 | 3 | 0 |
| 63 | Male | 39 | NSCLC | III | 2019-09-23 | 2 | 2 | 0 | 0 |
| 64 | Male | 52 | NSCLC | III | 2019-09-23 | 54 | 18 | 15 | 21 |
| 65 | Male | 49 | NSCLC | III | 2019-09-26 | 17 | 9 | 6 | 2 |
| 66 | Female | 59 | NSCLC | III | 2019-09-26 | 24 | 10 | 10 | 4 |
| 67 | Female | 54 | NSCLC | III | 2019-09-29 | 22 | 10 | 8 | 4 |
| 68 | Female | 55 | NSCLC | III | 2019-09-29 | 49 | 24 | 13 | 12 |
| 69 | Female | 51 | NSCLC | III | 2019-09-29 | 15 | 7 | 6 | 2 |
| 70 | Male | 59 | NSCLC | III | 2019-10-08 | 16 | 9 | 5 | 2 |
| 71 | Female | 35 | NSCLC | III | 2019-10-12 | 12 | 6 | 4 | 2 |
| 72 | Female | 38 | NSCLC | III | 2019-10-12 | 54 | 25 | 14 | 15 |
| 73 | Female | 41 | NSCLC | III | 2019-10-12 | 17 | 12 | 5 | 0 |
| 74 | Female | 50 | NSCLC | III | 2019-10-29 | 24 | 12 | 6 | 6 |
| 75 | Female | 50 | NSCLC | III | 2019-10-29 | 20 | 13 | 5 | 2 |
| 76 | Female | 63 | NSCLC | III | 2019-10-29 | 49 | 23 | 19 | 7 |
| 77 | Male | 54 | NSCLC | III | 2019-10-29 | 15 | 5 | 8 | 2 |
| 78 | Female | 49 | NSCLC | III | 2019-10-29 | 30 | 16 | 10 | 4 |
| 79 | Male | 54 | NSCLC | III | 2019-10-29 | 24 | 10 | 8 | 6 |
| 80 | Female | 49 | NSCLC | III | 2019-10-29 | 22 | 10 | 9 | 3 |
| 81 | Female | 63 | NSCLC | III | 2019-10-29 | 49 | 23 | 20 | 6 |
| 82 | Female | 49 | NSCLC | III | 2019-10-29 | 15 | 9 | 5 | 1 |
| 83 | Female | 50 | NSCLC | III | 2019-10-29 | 30 | 16 | 14 | 0 |
| 84 | Female | 63 | NSCLC | III | 2019-10-29 | 12 | 6 | 5 | 1 |
| 85 | Male | 89 | NSCLC | III | 2019-10-30 | 46 | 27 | 19 | 0 |
| 86 | Male | 50 | NSCLC | III | 2019-10-30 | 33 | 14 | 13 | 6 |
| 87 | Male | 52 | NSCLC | IV | 2019-11-05 | 24 | 12 | 5 | 7 |
| 88 | Female | 61 | NSCLC | IV | 2019-11-05 | 19 | 11 | 5 | 3 |
| 89 | Female | 64 | NSCLC | IV | 2019-11-05 | 17 | 9 | 8 | 0 |
| 90 | Female | 60 | NSCLC | IV | 2019-11-06 | 24 | 8 | 8 | 8 |
| 91 | Female | 60 | NSCLC | IV | 2019-11-06 | 22 | 10 | 5 | 7 |
| 92 | Female | 64 | NSCLC | IV | 2019-11-10 | 49 | 26 | 17 | 6 |
| 93 | Male | 63 | NSCLC | IV | 2019-11-10 | 31 | 14 | 13 | 4 |
| 94 | Female | 61 | NSCLC | IV | 2019-11-10 | 19 | 10 | 3 | 6 |
| 95 | Male | 61 | NSCLC | IV | 2019-11-10 | 24 | 12 | 10 | 2 |
| 96 | Female | 46 | NSCLC | IV | 2019-11-14 | 22 | 11 | 10 | 1 |
| 97 | Female | 47 | NSCLC | IV | 2019-11-14 | 49 | 23 | 17 | 9 |
| 98 | Female | 55 | NSCLC | IV | 2019-11-14 | 28 | 13 | 10 | 5 |
| 99 | Female | 37 | NSCLC | IV | 2019-11-15 | 16 | 12 | 3 | 1 |
| 100 | Female | 62 | NSCLC | IV | 2019-11-15 | 25 | 13 | 10 | 2 |
| 101 | Female | 64 | NSCLC | IV | 2019-11-15 | 15 | 8 | 5 | 2 |
| 102 | Female | 39 | NSCLC | IV | 2019-11-18 | 16 | 6 | 8 | 2 |
| 103 | Female | 52 | NSCLC | IV | 2019-11-21 | 24 | 12 | 7 | 5 |
| 104 | Female | 32 | NSCLC | IV | 2019-11-22 | 22 | 13 | 6 | 3 |
| 105 | Female | 44 | NSCLC | IV | 2019-11-23 | 44 | 26 | 13 | 5 |
| 106 | Female | 51 | NSCLC | IV | 2019-11-27 | 15 | 10 | 4 | 1 |
| 107 | Female | 70 | NSCLC | IV | 2019-11-27 | 29 | 16 | 13 | 0 |
| 108 | Male | 56 | NSCLC | IV | 2019-11-27 | 12 | 6 | 4 | 2 |
| 109 | Female | 32 | NSCLC | IV | 2019-11-27 | 48 | 22 | 21 | 5 |
| 110 | Female | 35 | NSCLC | IV | 2019-11-28 | 17 | 9 | 6 | 2 |
| 111 | Female | 77 | NSCLC | IV | 2019-11-28 | 24 | 11 | 4 | 9 |
| 112 | Female | 59 | NSCLC | IV | 2019-11-28 | 29 | 11 | 10 | 8 |
| 113 | Female | 60 | NSCLC | IV | 2019-11-28 | 22 | 10 | 11 | 1 |
| 114 | Female | 60 | NSCLC | IV | 2019-11-28 | 21 | 9 | 9 | 3 |
| 115 | Female | 40 | NSCLC | IV | 2019-11-29 | 22 | 6 | 8 | 8 |
| 116 | Female | 49 | NSCLC | IV | 2019-11-29 | 52 | 27 | 18 | 7 |
| 117 | Female | 48 | NSCLC | IV | 2019-11-29 | 15 | 5 | 5 | 5 |
| 118 | Female | 62 | NSCLC | IV | 2019-12-01 | 16 | 4 | 5 | 7 |
| 119 | Female | 37 | NSCLC | IV | 2019-12-01 | 24 | 8 | 9 | 7 |
| 120 | Female | 53 | NSCLC | IV | 2019-12-03 | 18 | 7 | 6 | 5 |
| 121 | Male | 54 | NSCLC | IV | 2019-12-03 | 49 | 21 | 19 | 9 |
| 122 | Male | 45 | NSCLC | IV | 2019-12-03 | 24 | 15 | 7 | 2 |
| 123 | Female | 43 | NSCLC | IV | 2019-12-03 | 22 | 10 | 5 | 7 |
| 124 | Female | 55 | NSCLC | IV | 2019-12-05 | 49 | 26 | 13 | 10 |
| 125 | Female | 65 | NSCLC | IV | 2019-12-05 | 22 | 6 | 7 | 9 |
| 126 | Female | 42 | NSCLC | IV | 2019-12-05 | 16 | 5 | 9 | 2 |
| 127 | Female | 46 | NSCLC | IV | 2019-12-05 | 24 | 5 | 8 | 11 |
| 128 | Male | 69 | NSCLC | IV | 2019-12-05 | 22 | 6 | 4 | 12 |
| 129 | Female | 61 | BC | I | 2019-12-05 | 3 | 1 | 1 | 1 |
| 130 | Female | 45 | BC | I | 2019-12-06 | 9 | 6 | 3 | 0 |
| 131 | Female | 52 | BC | I | 2019-12-06 | 7 | 5 | 2 | 0 |
| 132 | Female | 53 | BC | I | 2019-12-06 | 5 | 3 | 1 | 1 |
| 133 | Female | 57 | BC | I | 2019-12-08 | 14 | 5 | 6 | 3 |
| 134 | Female | 52 | BC | I | 2019-12-08 | 10 | 6 | 2 | 2 |
| 135 | Female | 66 | BC | I | 2019-12-04 | 3 | 1 | 1 | 1 |
| 136 | Female | 76 | BC | I | 2019-12-10 | 1 | 1 | 0 | 0 |
| 137 | Female | 35 | BC | I | 2019-12-11 | 8 | 4 | 2 | 2 |
| 138 | Female | 60 | BC | I | 2019-12-11 | 4 | 2 | 1 | 1 |
| 139 | Female | 44 | BC | I | 2019-12-11 | 0 | 0 | 0 | 0 |
| 140 | Female | 62 | BC | I | 2019-12-12 | 4 | 2 | 1 | 1 |
| 141 | Female | 69 | BC | I | 2019-12-13 | 15 | 6 | 6 | 3 |
| 142 | Female | 50 | BC | I | 2019-12-17 | 10 | 7 | 2 | 1 |
| 143 | Female | 33 | BC | I | 2019-12-17 | 4 | 2 | 2 | 0 |
| 144 | Female | 38 | BC | I | 2019-12-17 | 11 | 4 | 6 | 1 |
| 145 | Female | 33 | BC | I | 2019-12-17 | 11 | 6 | 5 | 0 |
| 146 | Female | 56 | BC | I | 2019-12-19 | 7 | 5 | 1 | 1 |
| 147 | Female | 49 | BC | I | 2019-12-19 | 4 | 2 | 1 | 1 |
| 148 | Female | 65 | BC | I | 2019-12-19 | 3 | 1 | 0 | 2 |
| 149 | Female | 49 | BC | I | 2019-12-20 | 7 | 2 | 3 | 2 |
| 150 | Female | 52 | BC | I | 2019-12-20 | 8 | 5 | 1 | 2 |
| 151 | Female | 70 | BC | I | 2019-12-21 | 4 | 2 | 1 | 1 |
| 152 | Female | 50 | BC | I | 2019-12-23 | 4 | 1 | 1 | 2 |
| 153 | Female | 66 | BC | I | 2019-12-25 | 5 | 2 | 2 | 1 |
| 154 | Female | 58 | BC | I | 2019-12-25 | 3 | 2 | 1 | 0 |
| 155 | Female | 55 | BC | I | 2019-12-25 | 4 | 0 | 2 | 2 |
| 156 | Female | 58 | BC | I | 2019-12-25 | 4 | 2 | 2 | 0 |
| 157 | Female | 45 | BC | I | 2019-12-26 | 4 | 3 | 1 | 0 |
| 158 | Female | 46 | BC | I | 2019-12-26 | 9 | 5 | 2 | 2 |
| 159 | Female | 69 | BC | I | 2019-12-26 | 4 | 2 | 1 | 1 |
| 160 | Female | 74 | BC | I | 2019-12-26 | 1 | 0 | 1 | 0 |
| 161 | Female | 52 | BC | I | 2019-12-26 | 5 | 3 | 2 | 0 |
| 162 | Female | 56 | BC | I | 2019-12-26 | 2 | 0 | 2 | 0 |
| 163 | Female | 51 | BC | I | 2019-12-27 | 4 | 1 | 2 | 1 |
| 164 | Female | 49 | BC | I | 2019-12-27 | 3 | 1 | 1 | 1 |
| 165 | Female | 76 | BC | I | 2019-12-26 | 4 | 2 | 1 | 1 |
| 166 | Female | 42 | BC | I | 2019-12-31 | 4 | 2 | 0 | 2 |
| 167 | Female | 45 | BC | I | 2020-01-03 | 7 | 3 | 3 | 1 |
| 168 | Female | 57 | BC | I | 2020-01-06 | 3 | 2 | 1 | 0 |
| 169 | Female | 73 | BC | II | 2020-01-07 | 1 | 0 | 0 | 1 |
| 170 | Female | 49 | BC | II | 2020-01-07 | 4 | 1 | 1 | 2 |
| 171 | Female | 48 | BC | II | 2020-01-07 | 3 | 0 | 2 | 1 |
| 172 | Female | 53 | BC | II | 2020-01-09 | 4 | 3 | 1 | 0 |
| 173 | Female | 40 | BC | II | 2020-01-09 | 4 | 2 | 2 | 0 |
| 174 | Female | 51 | BC | II | 2020-01-10 | 6 | 1 | 1 | 4 |
| 175 | Female | 54 | BC | II | 2020-01-13 | 9 | 5 | 4 | 0 |
| 176 | Female | 66 | BC | II | 2020-01-13 | 5 | 3 | 2 | 0 |
| 177 | Female | 41 | BC | II | 2020-01-13 | 7 | 2 | 2 | 3 |
| 178 | Female | 29 | BC | II | 2020-01-14 | 0 | 0 | 0 | 0 |
| 179 | Female | 56 | BC | II | 2020-01-15 | 8 | 3 | 3 | 2 |
| 180 | Female | 47 | BC | II | 2020-01-16 | 11 | 5 | 2 | 4 |
| 181 | Female | 50 | BC | II | 2020-01-17 | 9 | 3 | 4 | 2 |
| 182 | Female | 42 | BC | II | 2020-02-18 | 14 | 3 | 5 | 6 |
| 183 | Female | 65 | BC | II | 2020-02-19 | 4 | 2 | 1 | 1 |
| 184 | Female | 63 | BC | II | 2020-03-06 | 6 | 2 | 3 | 1 |
| 185 | Female | 32 | BC | II | 2020-03-06 | 8 | 5 | 2 | 1 |
| 186 | Female | 74 | BC | II | 2020-03-06 | 17 | 8 | 7 | 2 |
| 187 | Female | 52 | BC | II | 2020-03-06 | 10 | 3 | 6 | 1 |
| 188 | Female | 61 | BC | II | 2020-03-09 | 20 | 8 | 6 | 6 |
| 189 | Female | 36 | BC | II | 2020-03-12 | 4 | 2 | 1 | 1 |
| 190 | Female | 43 | BC | II | 2020-03-12 | 18 | 9 | 8 | 1 |
| 191 | Female | 62 | BC | II | 2020-03-13 | 7 | 3 | 2 | 2 |
| 192 | Female | 61 | BC | II | 2020-03-16 | 4 | 2 | 0 | 2 |
| 193 | Female | 51 | BC | II | 2020-03-16 | 18 | 9 | 5 | 4 |
| 194 | Female | 50 | BC | II | 2020-03-18 | 30 | 12 | 13 | 5 |
| 195 | Female | 57 | BC | II | 2020-03-19 | 7 | 2 | 3 | 2 |
| 196 | Female | 56 | BC | II | 2020-03-20 | 3 | 2 | 0 | 1 |
| 197 | Female | 72 | BC | II | 2020-03-20 | 30 | 13 | 8 | 9 |
| 198 | Female | 50 | BC | II | 2020-03-26 | 20 | 10 | 8 | 2 |
| 199 | Female | 63 | BC | II | 2020-03-26 | 2 | 1 | 1 | 0 |
| 200 | Female | 52 | BC | II | 2020-03-27 | 6 | 3 | 1 | 2 |
| 201 | Female | 50 | BC | II | 2020-03-27 | 25 | 9 | 10 | 6 |
| 202 | Female | 54 | BC | II | 2020-03-27 | 12 | 5 | 5 | 2 |
| 203 | Female | 32 | BC | II | 2020-03-28 | 17 | 6 | 7 | 4 |
| 204 | Female | 37 | BC | II | 2020-03-29 | 4 | 2 | 1 | 1 |
| 205 | Female | 57 | BC | II | 2020-03-30 | 2 | 2 | 0 | 0 |
| 206 | Female | 49 | BC | II | 2020-03-31 | 3 | 1 | 1 | 1 |
| 207 | Female | 47 | BC | II | 2020-03-31 | 5 | 3 | 2 | 0 |
| 208 | Female | 33 | BC | II | 2020-03-31 | 2 | 1 | 0 | 1 |
| 209 | Female | 50 | BC | II | 2020-04-01 | 1 | 1 | 0 | 0 |
| 210 | Female | 52 | BC | II | 2020-04-01 | 4 | 2 | 2 | 0 |
| 211 | Female | 41 | BC | II | 2020-04-01 | 11 | 3 | 6 | 2 |
| 212 | Female | 46 | BC | II | 2020-04-02 | 0 | 0 | 0 | 0 |
| 213 | Female | 46 | BC | II | 2020-04-02 | 9 | 3 | 3 | 3 |
| 214 | Female | 44 | BC | II | 2020-04-02 | 2 | 2 | 0 | 0 |
| 215 | Female | 55 | BC | II | 2020-04-02 | 10 | 5 | 5 | 0 |
| 216 | Female | 40 | BC | II | 2020-04-02 | 6 | 2 | 3 | 1 |
| 217 | Female | 49 | BC | II | 2020-04-02 | 0 | 0 | 0 | 0 |
| 218 | Female | 62 | BC | II | 2020-04-03 | 6 | 3 | 3 | 0 |
| 219 | Female | 48 | BC | II | 2020-04-03 | 0 | 0 | 0 | 0 |
| 220 | Female | 31 | BC | II | 2020-04-07 | 6 | 3 | 3 | 0 |
| 221 | Female | 31 | BC | II | 2020-04-07 | 6 | 3 | 3 | 0 |
| 222 | Female | 59 | BC | II | 2020-04-07 | 17 | 9 | 5 | 3 |
| 223 | Female | 59 | BC | II | 2020-04-07 | 8 | 4 | 3 | 1 |
| 224 | Female | 65 | BC | II | 2020-04-07 | 24 | 10 | 12 | 2 |
| 225 | Female | 57 | BC | III | 2020-04-10 | 11 | 5 | 5 | 1 |
| 226 | Female | 47 | BC | III | 2020-04-10 | 23 | 13 | 8 | 2 |
| 227 | Female | 55 | BC | III | 2020-04-14 | 4 | 2 | 1 | 1 |
| 228 | Female | 27 | BC | III | 2020-04-14 | 2 | 0 | 2 | 0 |
| 229 | Female | 50 | BC | III | 2020-04-15 | 33 | 16 | 9 | 8 |
| 230 | Female | 36 | BC | III | 2020-04-15 | 14 | 5 | 6 | 3 |
| 231 | Female | 40 | BC | III | 2020-04-16 | 4 | 2 | 1 | 1 |
| 232 | Female | 33 | BC | III | 2020-04-16 | 14 | 9 | 5 | 0 |
| 233 | Female | 44 | BC | III | 2020-04-17 | 4 | 2 | 1 | 1 |
| 234 | Female | 42 | BC | III | 2020-04-17 | 11 | 6 | 5 | 0 |
| 235 | Female | 29 | BC | III | 2020-04-17 | 28 | 13 | 8 | 7 |
| 236 | Female | 64 | BC | III | 2020-04-17 | 5 | 3 | 2 | 0 |
| 237 | Female | 47 | BC | III | 2020-04-20 | 0 | 0 | 0 | 0 |
| 238 | Female | 40 | BC | III | 2020-04-21 | 0 | 0 | 0 | 0 |
| 239 | Female | 52 | BC | III | 2020-04-21 | 8 | 5 | 2 | 1 |
| 240 | Female | 49 | BC | III | 2020-04-21 | 0 | 0 | 0 | 0 |
| 241 | Female | 72 | BC | III | 2020-04-22 | 6 | 3 | 2 | 1 |
| 242 | Female | 48 | BC | III | 2020-04-23 | 120 | 38 | 45 | 37 |
| 243 | Female | 49 | BC | III | 2020-04-24 | 0 | 0 | 0 | 0 |
| 244 | Female | 56 | BC | III | 2020-04-27 | 26 | 13 | 6 | 7 |
| 245 | Female | 45 | BC | III | 2020-04-28 | 35 | 10 | 12 | 13 |
| 246 | Female | 32 | BC | III | 2020-05-06 | 6 | 2 | 3 | 1 |
| 247 | Female | 52 | BC | III | 2020-05-07 | 19 | 6 | 7 | 6 |
| 248 | Female | 51 | BC | III | 2020-05-07 | 31 | 15 | 6 | 10 |
| 249 | Female | 29 | BC | III | 2020-05-07 | 26 | 13 | 12 | 1 |
| 250 | Female | 59 | BC | III | 2020-05-08 | 23 | 12 | 10 | 1 |
| 251 | Female | 39 | BC | III | 2020-05-09 | 4 | 2 | 2 | 0 |
| 252 | Female | 52 | BC | III | 2020-05-09 | 17 | 8 | 7 | 2 |
| 253 | Female | 36 | BC | III | 2020-05-13 | 4 | 2 | 0 | 2 |
| 254 | Female | 32 | BC | III | 2020-05-12 | 0 | 0 | 0 | 0 |
| 255 | Female | 53 | BC | IV | 2020-05-14 | 8 | 3 | 1 | 4 |
| 256 | Female | 45 | BC | IV | 2020-05-14 | 14 | 7 | 3 | 4 |
| 257 | Female | 55 | BC | IV | 2020-05-13 | 22 | 11 | 6 | 5 |
| 258 | Female | 63 | BC | IV | 2020-05-19 | 3 | 2 | 1 | 0 |
| 259 | Female | 41 | BC | IV | 2020-05-19 | 4 | 0 | 2 | 2 |
| 260 | Female | 41 | BC | IV | 2020-05-19 | 11 | 4 | 6 | 1 |
| 261 | Female | 56 | BC | IV | 2020-05-19 | 36 | 13 | 15 | 8 |
| 262 | Female | 44 | BC | IV | 2020-05-19 | 8 | 2 | 5 | 1 |
| 263 | Female | 29 | BC | IV | 2020-05-22 | 20 | 9 | 8 | 3 |
| 264 | Female | 29 | BC | IV | 2020-05-22 | 44 | 12 | 19 | 13 |
| 265 | Female | 57 | BC | IV | 2020-05-25 | 26 | 13 | 10 | 3 |
| 266 | Female | 52 | BC | IV | 2020-05-26 | 12 | 10 | 2 | 0 |
| 267 | Female | 46 | BC | IV | 2020-05-29 | 12 | 6 | 5 | 1 |
| 268 | Female | 58 | BC | IV | 2020-05-29 | 42 | 18 | 19 | 5 |
| 269 | Female | 52 | BC | IV | 2020-05-29 | 34 | 17 | 16 | 1 |
| 270 | Female | 52 | BC | IV | 2020-06-02 | 12 | 6 | 4 | 2 |
| 271 | Female | 51 | BC | IV | 2020-06-02 | 5 | 2 | 2 | 1 |
| 272 | Female | 43 | BC | IV | 2020-06-01 | 27 | 14 | 11 | 2 |
| 273 | Female | 30 | BC | IV | 2020-06-03 | 240 | 112 | 109 | 19 |
| 274 | Female | 53 | BC | IV | 2020-06-02 | 3 | 2 | 1 | 0 |
| 275 | Female | 68 | BC | IV | 2020-06-02 | 27 | 15 | 9 | 3 |
| 276 | Female | 32 | BC | IV | 2020-06-02 | 18 | 8 | 8 | 2 |
| 277 | Female | 60 | BC | IV | 2020-06-04 | 13 | 6 | 3 | 4 |
| 278 | Female | 60 | BC | IV | 2020-06-04 | 23 | 15 | 8 | 0 |
| 279 | Female | 57 | BC | IV | 2020-06-04 | 8 | 2 | 2 | 4 |
| 280 | Female | 46 | BC | IV | 2020-06-04 | 8 | 1 | 3 | 4 |
| 281 | Female | 35 | BC | IV | 2020-04-24 | 15 | 5 | 6 | 4 |
| 282 | Female | 38 | BC | IV | 2020-06-04 | 36 | 18 | 13 | 5 |
| 283 | Female | 57 | BC | IV | 2020-06-05 | 7 | 2 | 3 | 2 |
| 284 | Female | 47 | BC | IV | 2020-06-05 | 7 | 2 | 2 | 3 |
| 285 | Female | 44 | BC | IV | 2020-06-09 | 29 | 19 | 5 | 5 |
| 286 | Female | 47 | BC | IV | 2020-06-09 | 4 | 2 | 2 | 0 |
| 287 | Female | 55 | BC | IV | 2020-06-09 | 55 | 26 | 18 | 11 |
| 288 | Female | 69 | BC | IV | 2020-06-09 | 2 | 1 | 1 | 0 |
| 289 | Female | 69 | BC | IV | 2020-06-09 | 520 | 210 | 201 | 109 |
| 290 | Male | 92 | PC | I | 2020-06-10 | 6 | 2 | 3 | 1 |
| 291 | Male | 81 | PC | I | 2020-06-10 | 0 | 0 | 0 | 0 |
| 292 | Male | 64 | PC | I | 2020-06-10 | 5 | 2 | 3 | 0 |
| 293 | Male | 92 | PC | I | 2020-06-10 | 9 | 3 | 2 | 4 |
| 294 | Male | 89 | PC | I | 2020-06-10 | 4 | 2 | 2 | 0 |
| 295 | Male | 85 | PC | I | 2020-06-10 | 3 | 1 | 2 | 0 |
| 296 | Male | 71 | PC | I | 2020-06-12 | 0 | 0 | 0 | 0 |
| 297 | Male | 64 | PC | I | 2020-06-12 | 3 | 1 | 1 | 1 |
| 298 | Male | 88 | PC | I | 2020-06-12 | 2 | 1 | 1 | 0 |
| 299 | Male | 80 | PC | I | 2020-06-12 | 16 | 10 | 3 | 3 |
| 300 | Male | 74 | PC | I | 2020-06-12 | 3 | 1 | 1 | 1 |
| 301 | Male | 70 | PC | I | 2020-06-12 | 5 | 2 | 1 | 2 |
| 302 | Male | 66 | PC | I | 2020-06-15 | 2 | 1 | 1 | 0 |
| 303 | Male | 85 | PC | I | 2020-06-14 | 9 | 6 | 2 | 1 |
| 304 | Male | 85 | PC | I | 2020-06-15 | 15 | 10 | 3 | 2 |
| 305 | Male | 70 | PC | I | 2020-06-17 | 8 | 5 | 3 | 0 |
| 306 | Male | 87 | PC | I | 2020-06-17 | 5 | 2 | 2 | 1 |
| 307 | Male | 78 | PC | I | 2020-06-22 | 3 | 2 | 1 | 0 |
| 308 | Male | 91 | PC | I | 2020-06-22 | 8 | 5 | 1 | 2 |
| 309 | Male | 61 | PC | I | 2020-06-22 | 24 | 13 | 6 | 5 |
| 310 | Male | 75 | PC | I | 2020-06-22 | 7 | 3 | 2 | 2 |
| 311 | Male | 93 | PC | I | 2020-06-23 | 32 | 16 | 14 | 2 |
| 312 | Male | 75 | PC | I | 2020-06-23 | 0 | 0 | 0 | 0 |
| 313 | Male | 94 | PC | I | 2020-06-23 | 5 | 3 | 2 | 0 |
| 314 | Male | 62 | PC | I | 2020-06-23 | 25 | 13 | 10 | 2 |
| 315 | Male | 86 | PC | I | 2020-06-23 | 3 | 1 | 2 | 0 |
| 316 | Male | 81 | PC | I | 2020-06-23 | 2 | 1 | 1 | 0 |
| 317 | Male | 88 | PC | I | 2020-06-23 | 28 | 15 | 13 | 0 |
| 318 | Male | 68 | PC | I | 2020-06-24 | 1 | 1 | 0 | 0 |
| 319 | Male | 85 | PC | I | 2020-06-24 | 23 | 10 | 11 | 2 |
| 320 | Male | 76 | PC | I | 2020-06-28 | 8 | 3 | 3 | 2 |
| 321 | Male | 85 | PC | I | 2020-06-29 | 14 | 7 | 4 | 3 |
| 322 | Male | 72 | PC | I | 2020-06-29 | 11 | 3 | 3 | 5 |
| 323 | Male | 89 | PC | I | 2020-06-29 | 16 | 5 | 6 | 5 |
| 324 | Male | 64 | PC | I | 2020-06-30 | 8 | 5 | 2 | 1 |
| 325 | Male | 65 | PC | I | 2020-06-30 | 4 | 2 | 2 | 0 |
| 326 | Male | 67 | PC | I | 2020-07-01 | 8 | 4 | 3 | 1 |
| 327 | Male | 76 | PC | I | 2020-07-02 | 14 | 3 | 6 | 5 |
| 328 | Male | 63 | PC | I | 2020-07-02 | 20 | 10 | 8 | 2 |
| 329 | Male | 77 | PC | II | 2020-07-02 | 7 | 3 | 2 | 2 |
| 330 | Male | 84 | PC | II | 2020-07-03 | 4 | 2 | 1 | 1 |
| 331 | Male | 61 | PC | II | 2020-07-02 | 5 | 2 | 3 | 0 |
| 332 | Male | 83 | PC | II | 2020-07-06 | 11 | 5 | 6 | 0 |
| 333 | Male | 89 | PC | II | 2020-07-06 | 35 | 12 | 10 | 13 |
| 334 | Male | 88 | PC | II | 2020-07-09 | 9 | 6 | 2 | 1 |
| 335 | Male | 76 | PC | II | 2020-07-09 | 8 | 2 | 3 | 3 |
| 336 | Male | 71 | PC | II | 2020-07-09 | 10 | 6 | 2 | 2 |
| 337 | Male | 83 | PC | II | 2020-07-10 | 1 | 1 | 0 | 0 |
| 338 | Male | 65 | PC | II | 2020-07-10 | 3 | 1 | 2 | 0 |
| 339 | Male | 85 | PC | II | 2020-07-10 | 30 | 13 | 12 | 5 |
| 340 | Male | 94 | PC | II | 2020-07-10 | 21 | 10 | 6 | 5 |
| 341 | Male | 88 | PC | II | 2020-07-10 | 2 | 1 | 0 | 1 |
| 342 | Male | 62 | PC | II | 2020-07-14 | 8 | 5 | 2 | 1 |
| 343 | Male | 65 | PC | II | 2020-07-15 | 4 | 2 | 1 | 1 |
| 344 | Male | 81 | PC | II | 2020-07-15 | 8 | 4 | 2 | 2 |
| 345 | Male | 76 | PC | II | 2020-07-15 | 25 | 10 | 12 | 3 |
| 346 | Male | 87 | PC | II | 2020-07-14 | 35 | 13 | 10 | 12 |
| 347 | Male | 81 | PC | II | 2020-07-15 | 16 | 6 | 8 | 2 |
| 348 | Male | 72 | PC | II | 2020-07-15 | 29 | 12 | 11 | 6 |
| 349 | Male | 69 | PC | II | 2020-07-17 | 23 | 6 | 9 | 8 |
| 350 | Male | 91 | PC | II | 2020-07-17 | 11 | 3 | 3 | 5 |
| 351 | Male | 85 | PC | II | 2020-07-17 | 9 | 5 | 2 | 2 |
| 352 | Male | 74 | PC | II | 2020-07-17 | 23 | 10 | 9 | 4 |
| 353 | Male | 89 | PC | II | 2020-07-17 | 27 | 12 | 10 | 5 |
| 354 | Male | 69 | PC | II | 2020-07-17 | 7 | 3 | 3 | 1 |
| 355 | Male | 76 | PC | II | 2020-07-17 | 0 | 0 | 0 | 0 |
| 356 | Male | 63 | PC | II | 2020-07-17 | 0 | 0 | 0 | 0 |
| 357 | Male | 75 | PC | II | 2020-07-18 | 0 | 0 | 0 | 0 |
| 358 | Male | 90 | PC | II | 2020-07-20 | 3 | 2 | 1 | 0 |
| 359 | Male | 83 | PC | II | 2020-07-19 | 13 | 6 | 5 | 2 |
| 360 | Male | 86 | PC | II | 2020-07-20 | 26 | 16 | 8 | 2 |
| 361 | Male | 88 | PC | II | 2020-07-21 | 29 | 14 | 7 | 8 |
| 362 | Male | 79 | PC | II | 2020-07-22 | 10 | 3 | 5 | 2 |
| 363 | Male | 72 | PC | II | 2020-07-22 | 13 | 7 | 2 | 4 |
| 364 | Male | 83 | PC | II | 2020-07-23 | 5 | 2 | 1 | 2 |
| 365 | Male | 84 | PC | II | 2020-07-23 | 57 | 24 | 18 | 15 |
| 366 | Male | 61 | PC | II | 2020-07-24 | 29 | 12 | 7 | 10 |
| 367 | Male | 89 | PC | II | 2020-07-24 | 10 | 6 | 3 | 1 |
| 368 | Male | 94 | PC | II | 2020-07-24 | 22 | 10 | 8 | 4 |
| 369 | Male | 92 | PC | II | 2020-07-24 | 12 | 5 | 6 | 1 |
| 370 | Male | 92 | PC | II | 2020-07-24 | 6 | 3 | 3 | 0 |
| 371 | Male | 76 | PC | II | 2020-07-24 | 11 | 2 | 3 | 6 |
| 372 | Male | 73 | PC | II | 2020-07-24 | 0 | 0 | 0 | 0 |
| 373 | Male | 68 | PC | II | 2020-07-24 | 8 | 4 | 2 | 2 |
| 374 | Male | 86 | PC | II | 2020-07-24 | 0 | 0 | 0 | 0 |
| 375 | Male | 75 | PC | II | 2020-07-24 | 0 | 0 | 0 | 0 |
| 376 | Male | 68 | PC | II | 2020-07-27 | 12 | 8 | 3 | 1 |
| 377 | Male | 75 | PC | II | 2020-07-28 | 12 | 5 | 6 | 1 |
| 378 | Male | 87 | PC | II | 2020-07-28 | 5 | 2 | 3 | 0 |
| 379 | Male | 91 | PC | II | 2020-07-30 | 7 | 2 | 3 | 2 |
| 380 | Male | 66 | PC | II | 2020-07-30 | 12 | 5 | 4 | 3 |
| 381 | Male | 64 | PC | II | 2020-07-30 | 9 | 6 | 3 | 0 |
| 382 | Male | 81 | PC | II | 2020-07-31 | 14 | 5 | 3 | 6 |
| 383 | Male | 76 | PC | II | 2020-07-31 | 10 | 5 | 2 | 3 |
| 384 | Male | 83 | PC | II | 2020-08-03 | 25 | 12 | 10 | 3 |
| 385 | Male | 75 | PC | II | 2020-08-03 | 6 | 3 | 2 | 1 |
| 386 | Male | 61 | PC | II | 2020-08-04 | 11 | 5 | 5 | 1 |
| 387 | Male | 84 | PC | II | 2020-08-04 | 12 | 6 | 2 | 4 |
| 388 | Male | 77 | PC | II | 2020-08-05 | 6 | 2 | 3 | 1 |
| 389 | Male | 78 | PC | II | 2020-08-05 | 14 | 5 | 6 | 3 |
| 390 | Male | 69 | PC | II | 2020-08-06 | 8 | 8 | 0 | 0 |
| 391 | Male | 66 | PC | III | 2020-08-06 | 10 | 4 | 5 | 1 |
| 392 | Male | 87 | PC | III | 2020-08-07 | 10 | 6 | 2 | 2 |
| 393 | Male | 69 | PC | III | 2020-08-08 | 9 | 3 | 3 | 3 |
| 394 | Male | 89 | PC | III | 2020-08-08 | 6 | 2 | 2 | 2 |
| 395 | Male | 62 | PC | III | 2020-08-08 | 4 | 2 | 1 | 1 |
| 396 | Male | 75 | PC | III | 2020-08-10 | 6 | 3 | 0 | 3 |
| 397 | Male | 65 | PC | III | 2020-08-10 | 120 | 56 | 39 | 25 |
| 398 | Male | 66 | PC | III | 2020-08-10 | 5 | 2 | 2 | 1 |
| 399 | Male | 93 | PC | III | 2020-08-10 | 4 | 1 | 1 | 2 |
| 400 | Male | 70 | PC | III | 2020-08-12 | 20 | 14 | 3 | 3 |
| 401 | Male | 75 | PC | III | 2020-08-12 | 24 | 5 | 8 | 11 |
| 402 | Male | 75 | PC | III | 2020-08-12 | 13 | 8 | 5 | 0 |
| 403 | Male | 76 | PC | III | 2020-08-12 | 19 | 5 | 10 | 4 |
| 404 | Male | 85 | PC | III | 2020-08-13 | 37 | 23 | 10 | 4 |
| 405 | Male | 94 | PC | III | 2020-08-13 | 49 | 15 | 10 | 24 |
| 406 | Male | 68 | PC | III | 2020-08-13 | 31 | 10 | 12 | 9 |
| 407 | Male | 72 | PC | III | 2020-08-13 | 24 | 9 | 9 | 6 |
| 408 | Male | 61 | PC | III | 2020-08-13 | 9 | 3 | 3 | 3 |
| 409 | Male | 81 | PC | III | 2020-08-17 | 7 | 3 | 3 | 1 |
| 410 | Male | 77 | PC | III | 2020-08-18 | 0 | 0 | 0 | 0 |
| 411 | Male | 89 | PC | III | 2020-08-18 | 22 | 6 | 7 | 9 |
| 412 | Male | 80 | PC | III | 2020-08-18 | 4 | 2 | 2 | 0 |
| 413 | Male | 91 | PC | III | 2020-08-18 | 13 | 5 | 6 | 2 |
| 414 | Male | 84 | PC | III | 2020-08-18 | 14 | 5 | 4 | 5 |
| 415 | Male | 70 | PC | III | 2020-08-18 | 12 | 6 | 2 | 4 |
| 416 | Male | 82 | PC | III | 2020-08-19 | 1 | 0 | 1 | 0 |
| 417 | Male | 89 | PC | III | 2020-08-20 | 15 | 6 | 9 | 0 |
| 418 | Male | 73 | PC | III | 2020-08-20 | 9 | 3 | 6 | 0 |
| 419 | Male | 64 | PC | III | 2020-08-20 | 4 | 1 | 2 | 1 |
| 420 | Male | 84 | PC | IV | 2020-08-20 | 150 | 64 | 69 | 17 |
| 421 | Male | 70 | PC | IV | 2020-08-20 | 13 | 3 | 5 | 5 |
| 422 | Male | 88 | PC | IV | 2020-08-20 | 2 | 1 | 1 | 0 |
| 423 | Male | 93 | PC | IV | 2020-08-21 | 94 | 56 | 20 | 18 |
| 424 | Male | 88 | PC | IV | 2020-08-21 | 8 | 3 | 5 | 0 |
| 425 | Male | 85 | PC | IV | 2020-08-24 | 0 | 0 | 0 | 0 |
| 426 | Male | 74 | PC | IV | 2020-08-24 | 13 | 5 | 3 | 5 |
| 427 | Male | 72 | PC | IV | 2020-08-24 | 4 | 2 | 2 | 0 |
| 428 | Male | 62 | PC | IV | 2020-08-24 | 7 | 3 | 3 | 1 |
| 429 | Male | 88 | PC | IV | 2020-08-24 | 9 | 3 | 2 | 4 |
| 430 | Male | 94 | PC | IV | 2020-08-25 | 6 | 3 | 3 | 0 |
| 431 | Male | 88 | PC | IV | 2020-08-25 | 4 | 2 | 0 | 2 |
| 432 | Male | 88 | PC | IV | 2020-08-25 | 150 | 67 | 56 | 27 |
| 433 | Male | 91 | PC | IV | 2020-08-25 | 13 | 5 | 6 | 2 |
| 434 | Male | 78 | PC | IV | 2020-08-25 | 9 | 3 | 2 | 4 |
| 435 | Male | 73 | PC | IV | 2020-08-26 | 180 | 75 | 68 | 37 |
| 436 | Male | 90 | PC | IV | 2020-08-27 | 22 | 9 | 8 | 5 |
| 437 | Male | 71 | PC | IV | 2020-08-27 | 5 | 2 | 1 | 2 |
| 438 | Male | 91 | PC | IV | 2020-08-27 | 11 | 5 | 2 | 4 |
| 439 | Male | 71 | PC | IV | 2020-08-27 | 10 | 2 | 5 | 3 |
| 440 | Male | 86 | PC | IV | 2020-08-28 | 120 | 54 | 49 | 17 |
| 441 | Male | 68 | PC | IV | 2020-08-31 | 3 | 1 | 1 | 1 |
| 442 | Male | 69 | PC | IV | 2020-09-02 | 6 | 2 | 1 | 3 |
| 443 | Male | 79 | PC | IV | 2020-09-01 | 12 | 6 | 2 | 4 |
| 444 | Male | 62 | PC | IV | 2020-09-02 | 90 | 35 | 30 | 25 |
| 445 | Male | 90 | PC | IV | 2020-09-03 | 120 | 55 | 45 | 20 |
| 446 | Male | 63 | PC | IV | 2020-09-03 | 8 | 2 | 3 | 3 |
| 447 | Male | 75 | PC | IV | 2020-09-03 | 12 | 3 | 2 | 7 |
| 448 | Male | 71 | PC | IV | 2020-09-03 | 14 | 5 | 3 | 6 |
| 449 | Male | 82 | PC | IV | 2020-09-03 | 6 | 3 | 3 | 0 |
| 450 | Male | 85 | PC | IV | 2020-09-03 | 12 | 2 | 3 | 7 |
| 451 | Male | 61 | PC | IV | 2020-09-03 | 3 | 2 | 1 | 0 |
| 452 | Male | 86 | PC | IV | 2020-09-03 | 9 | 5 | 2 | 2 |
| 453 | Male | 73 | PC | IV | 2020-09-03 | 5 | 2 | 3 | 0 |
| 454 | Male | 81 | PC | IV | 2020-09-03 | 200 | 93 | 86 | 21 |
| 455 | Male | 62 | PC | IV | 2020-09-03 | 8 | 2 | 3 | 3 |
| 456 | Male | 73 | HCC | II | 2020-03-06 | 4 | 2 | 2 | 0 |
| 457 | Male | 86 | HCC | II | 2020-03-10 | 5 | 2 | 1 | 2 |
| 458 | Female | 74 | HCC | II | 2020-03-26 | 7 | 2 | 4 | 1 |
| 459 | Female | 76 | HCC | III | 2020-03-31 | 5 | 2 | 3 | 0 |
| 460 | Female | 86 | HCC | IV | 2020-09-20 | 6 | 3 | 1 | 2 |
| 461 | Female | 64 | PAAD | I | 2020-09-07 | 4 | 2 | 2 | 0 |
| 462 | Female | 64 | PAAD | I | 2020-09-07 | 5 | 2 | 3 | 0 |
| 463 | Male | 64 | PAAD | I | 2020-09-09 | 1 | 0 | 1 | 0 |
| 464 | Male | 62 | PAAD | I | 2020-09-10 | 5 | 3 | 2 | 0 |
| 465 | Female | 59 | PAAD | I | 2020-09-10 | 9 | 5 | 2 | 2 |
| 466 | Female | 64 | PAAD | I | 2020-09-10 | 6 | 0 | 4 | 2 |
| 467 | Female | 64 | PAAD | I | 2020-09-10 | 5 | 3 | 1 | 1 |
| 468 | Female | 58 | PAAD | I | 2020-09-10 | 2 | 1 | 0 | 1 |
| 469 | Female | 62 | PAAD | I | 2020-09-11 | 9 | 3 | 2 | 4 |
| 470 | Female | 66 | PAAD | I | 2020-09-11 | 6 | 2 | 3 | 1 |
| 471 | Male | 47 | PAAD | I | 2020-09-14 | 10 | 5 | 5 | 0 |
| 472 | Female | 68 | PAAD | I | 2020-09-14 | 1 | 0 | 1 | 0 |
| 473 | Male | 68 | PAAD | I | 2020-09-16 | 0 | 0 | 0 | 0 |
| 474 | Female | 67 | PAAD | I | 2020-09-16 | 15 | 9 | 3 | 3 |
| 475 | Male | 65 | PAAD | I | 2020-09-16 | 5 | 3 | 2 | 0 |
| 476 | Male | 79 | PAAD | I | 2020-09-16 | 4 | 2 | 1 | 1 |
| 477 | Male | 69 | PAAD | II | 2020-09-16 | 2 | 1 | 1 | 0 |
| 478 | Female | 72 | PAAD | II | 2020-09-15 | 23 | 10 | 5 | 8 |
| 479 | Male | 84 | PAAD | II | 2020-09-15 | 29 | 13 | 8 | 8 |
| 480 | Male | 51 | PAAD | II | 2020-09-15 | 8 | 5 | 3 | 0 |
| 481 | Female | 51 | PAAD | II | 2020-09-15 | 4 | 2 | 1 | 1 |
| 482 | Female | 64 | PAAD | II | 2020-09-18 | 1 | 1 | 0 | 0 |
| 483 | Female | 45 | PAAD | II | 2020-09-18 | 2 | 1 | 0 | 1 |
| 484 | Female | 50 | PAAD | II | 2020-09-18 | 0 | 0 | 0 | 0 |
| 485 | Female | 61 | PAAD | II | 2020-09-18 | 14 | 5 | 6 | 3 |
| 486 | Female | 62 | PAAD | II | 2020-09-18 | 2 | 1 | 1 | 0 |
| 487 | Female | 58 | PAAD | II | 2020-09-19 | 2 | 1 | 0 | 1 |
| 488 | Female | 71 | PAAD | II | 2020-09-19 | 11 | 5 | 6 | 0 |
| 489 | Female | 67 | PAAD | II | 2020-09-19 | 1 | 0 | 1 | 0 |
| 490 | Female | 76 | PAAD | II | 2020-09-19 | 5 | 1 | 2 | 2 |
| 491 | Female | 66 | PAAD | II | 2020-09-22 | 2 | 0 | 1 | 1 |
| 492 | Female | 77 | PAAD | III | 2020-09-22 | 1 | 1 | 0 | 0 |
| 493 | Female | 58 | PAAD | III | 2020-09-24 | 0 | 0 | 0 | 0 |
| 494 | Male | 75 | PAAD | III | 2020-09-24 | 8 | 4 | 2 | 2 |
| 495 | Female | 72 | PAAD | III | 2020-09-25 | 26 | 14 | 8 | 4 |
| 496 | Male | 70 | PAAD | III | 2020-09-25 | 44 | 13 | 23 | 8 |
| 497 | Female | 74 | PAAD | III | 2020-09-26 | 11 | 5 | 2 | 4 |
| 498 | Male | 68 | PAAD | IV | 2020-09-27 | 8 | 2 | 3 | 3 |
| 499 | Male | 59 | PAAD | IV | 2020-09-27 | 8 | 3 | 2 | 3 |
| 500 | Male | 73 | PAAD | IV | 2020-09-27 | 10 | 5 | 3 | 2 |
| 501 | Female | 71 | PAAD | IV | 2020-09-27 | 7 | 3 | 2 | 2 |
| 502 | Female | 74 | PAAD | IV | 2020-09-29 | 4 | 2 | 0 | 2 |
| 503 | Male | 54 | PAAD | IV | 2020-09-29 | 31 | 15 | 10 | 6 |
| 504 | 71 | 75 | PAAD | IV | 2020-09-29 | 8 | 5 | 2 | 1 |
| 505 | Female | 70 | PAAD | IV | 2020-10-10 | 120 | 56 | 51 | 13 |
| 506 | Female | 53 | PAAD | IV | 2020-10-10 | 3 | 1 | 1 | 1 |
| 507 | Female | 77 | PAAD | IV | 2020-10-10 | 7 | 2 | 3 | 2 |
| 508 | Female | 59 | PAAD | IV | 2020-10-10 | 17 | 3 | 5 | 9 |
| 509 | Male | 56 | PAAD | IV | 2020-10-10 | 13 | 5 | 2 | 6 |
| 510 | Female | 69 | PAAD | IV | 2020-10-10 | 12 | 5 | 3 | 4 |
| 511 | Male | 69 | PAAD | IV | 2020-10-09 | 24 | 12 | 5 | 7 |
| 512 | Female | 68 | PAAD | IV | 2020-10-12 | 8 | 4 | 2 | 2 |
| 513 | Male | 63 | PAAD | IV | 2020-10-12 | 150 | 56 | 71 | 23 |
| 514 | Male | 49 | EC | I | 2020-10-12 | 1 | 0 | 1 | 0 |
| 515 | Female | 58 | EC | I | 2020-10-12 | 7 | 2 | 3 | 2 |
| 516 | Male | 81 | EC | I | 2020-10-12 | 4 | 2 | 1 | 1 |
| 517 | Female | 72 | EC | I | 2020-10-13 | 2 | 1 | 0 | 1 |
| 518 | Male | 63 | EC | I | 2020-10-13 | 0 | 0 | 0 | 0 |
| 519 | Male | 48 | EC | I | 2020-10-13 | 8 | 4 | 3 | 1 |
| 520 | Male | 67 | EC | I | 2020-10-13 | 2 | 1 | 0 | 1 |
| 521 | Female | 63 | EC | I | 2020-10-13 | 24 | 13 | 7 | 4 |
| 522 | Male | 59 | EC | I | 2020-10-13 | 1 | 1 | 0 | 0 |
| 523 | Male | 62 | EC | I | 2020-10-14 | 2 | 2 | 0 | 0 |
| 524 | Male | 54 | EC | I | 2020-10-14 | 9 | 3 | 3 | 3 |
| 525 | Male | 66 | EC | I | 2020-10-14 | 1 | 0 | 1 | 0 |
| 526 | Male | 67 | EC | I | 2020-10-14 | 2 | 0 | 2 | 0 |
| 527 | Male | 79 | EC | II | 2020-10-13 | 2 | 0 | 2 | 0 |
| 528 | Female | 75 | EC | II | 2020-10-13 | 6 | 2 | 3 | 1 |
| 529 | Male | 69 | EC | II | 2020-10-13 | 3 | 0 | 2 | 1 |
| 530 | Female | 62 | EC | II | 2020-10-15 | 1 | 0 | 1 | 0 |
| 531 | Male | 78 | EC | II | 2020-10-15 | 6 | 2 | 3 | 1 |
| 532 | Male | 72 | EC | II | 2020-10-15 | 1 | 0 | 1 | 0 |
| 533 | Male | 69 | EC | II | 2020-10-15 | 11 | 2 | 3 | 6 |
| 534 | Female | 74 | EC | II | 2020-10-15 | 2 | 0 | 1 | 1 |
| 535 | Male | 74 | EC | II | 2020-10-15 | 2 | 1 | 1 | 0 |
| 536 | Male | 71 | EC | II | 2020-10-16 | 2 | 2 | 0 | 0 |
| 537 | Male | 75 | EC | II | 2020-10-16 | 2 | 1 | 1 | 0 |
| 538 | Female | 62 | EC | II | 2020-10-16 | 10 | 5 | 2 | 3 |
| 539 | Male | 64 | EC | II | 2020-10-16 | 3 | 1 | 2 | 0 |
| 540 | Male | 64 | EC | II | 2020-10-16 | 2 | 0 | 1 | 1 |
| 541 | Female | 77 | EC | II | 2020-10-16 | 3 | 2 | 1 | 0 |
| 542 | Male | 67 | EC | II | 2020-10-16 | 2 | 1 | 1 | 0 |
| 543 | Male | 49 | EC | II | 2020-10-17 | 2 | 0 | 1 | 1 |
| 544 | Male | 69 | EC | II | 2020-10-17 | 8 | 5 | 2 | 1 |
| 545 | Female | 74 | EC | II | 2020-10-16 | 16 | 5 | 4 | 7 |
| 546 | Male | 69 | EC | II | 2020-10-18 | 9 | 3 | 2 | 4 |
| 547 | Male | 75 | EC | II | 2020-10-16 | 4 | 2 | 1 | 1 |
| 548 | Male | 74 | EC | II | 2020-10-16 | 5 | 5 | 0 | 0 |
| 549 | Male | 76 | EC | II | 2020-10-19 | 9 | 5 | 2 | 2 |
| 550 | Male | 67 | EC | II | 2020-10-19 | 4 | 1 | 2 | 1 |
| 551 | Female | 66 | EC | II | 2020-10-19 | 7 | 3 | 1 | 3 |
| 552 | Female | 66 | EC | II | 2020-10-19 | 3 | 1 | 1 | 1 |
| 553 | Male | 69 | EC | III | 2020-10-19 | 3 | 2 | 1 | 0 |
| 554 | Male | 65 | EC | III | 2020-10-19 | 8 | 3 | 2 | 3 |
| 555 | Male | 73 | EC | III | 2020-10-19 | 4 | 2 | 1 | 1 |
| 556 | Male | 53 | EC | III | 2020-10-20 | 2 | 1 | 0 | 1 |
| 557 | Female | 74 | EC | III | 2020-10-20 | 8 | 2 | 3 | 3 |
| 558 | Male | 49 | EC | III | 2020-10-20 | 4 | 2 | 1 | 1 |
| 559 | Male | 77 | EC | III | 2020-10-20 | 2 | 1 | 1 | 0 |
| 560 | Female | 71 | EC | III | 2020-10-20 | 5 | 2 | 3 | 0 |
| 561 | Female | 62 | EC | III | 2020-10-20 | 4 | 2 | 1 | 1 |
| 562 | Male | 44 | EC | III | 2020-10-20 | 12 | 2 | 1 | 9 |
| 563 | Female | 43 | EC | III | 2020-10-20 | 5 | 3 | 1 | 1 |
| 564 | Male | 63 | EC | III | 2020-10-20 | 8 | 2 | 3 | 3 |
| 565 | Male | 76 | EC | III | 2020-10-20 | 12 | 5 | 3 | 4 |
| 566 | Male | 74 | EC | IV | 2020-10-20 | 4 | 2 | 1 | 1 |
| 567 | Female | 78 | EC | IV | 2020-10-20 | 18 | 8 | 7 | 3 |
| 568 | Female | 68 | EC | IV | 2020-10-20 | 19 | 6 | 9 | 4 |
| 569 | Male | 52 | EC | IV | 2020-10-20 | 25 | 10 | 6 | 9 |
| 570 | Male | 48 | EC | IV | 2020-10-21 | 5 | 2 | 2 | 1 |
| 571 | Male | 46 | EC | IV | 2020-10-20 | 4 | 1 | 2 | 1 |
| 572 | Female | 55 | EC | IV | 2020-10-23 | 9 | 3 | 2 | 4 |

**Supplementary Table 5.**  **Clinical information and CTC results of healthy subjects**

| Number | Sex | Age | Detection time | CTC count (/4mL) |
| --- | --- | --- | --- | --- |
| 1 | Male | 65 | 2019-07-19 | 2 |
| 2 | Male | 62 | 2019-07-25 | 0 |
| 3 | Female | 31 | 2019-07-26 | 0 |
| 4 | Male | 31 | 2019-09-04 | 1 |
| 5 | Female | 66 | 2019-09-09 | 0 |
| 6 | Male | 72 | 2019-09-23 | 0 |
| 7 | Female | 52 | 2019-11-21 | 0 |
| 8 | Male | 52 | 2019-12-09 | 3 |
| 9 | Female | 67 | 2020-03-06 | 0 |
| 10 | Male | 61 | 2020-03-10 | 1 |
| 11 | Male | 51 | 2020-04-15 | 0 |
| 12 | Female | 61 | 2020-04-15 | 2 |
| 13 | Male | 61 | 2020-04-21 | 6 |
| 14 | Male | 57 | 2020-04-21 | 0 |
| 15 | Male | 57 | 2020-05-08 | 3 |
| 16 | Female | 58 | 2020-06-10 | 1 |
| 17 | Female | 57 | 2020-06-13 | 0 |
| 18 | Male | 41 | 2020-06-13 | 0 |
| 19 | Female | 41 | 2020-07-09 | 1 |
| 20 | Male | 29 | 2020-08-28 | 1 |
| 21 | Male | 48 | 2020-09-10 | 1 |
| 22 | Female | 49 | 2020-09-24 | 0 |
| 23 | Female | 67 | 2020-10-13 | 1 |
| 24 | Male | 65 | 2020-10-25 | 0 |
| 25 | Female | 56 | 2020-10-26 | 1 |
| 26 | Male | 56 | 2020-10-29 | 0 |
| 27 | Male | 62 | 2020-10-29 | 0 |
| 28 | Female | 58 | 2020-11-02 | 0 |
| 29 | Male | 49 | 2020-11-04 | 1 |
| 30 | Female | 72 | 2020-11-06 | 1 |
| 31 | Male | 56 | 2020-11-09 | 1 |
| 32 | Female | 56 | 2020-11-15 | 0 |
| 33 | Female | 44 | 2020-11-15 | 1 |
| 34 | Female | 46 | 2020-11-16 | 0 |
| 35 | Male | 53 | 2020-11-24 | 1 |
| 36 | Female | 56 | 2020-11-25 | 0 |
| 37 | Male | 56 | 2020-11-26 | 2 |
| 38 | Male | 61 | 2020-12-04 | 0 |
| 39 | Female | 57 | 2020-12-04 | 3 |
| 40 | Female | 58 | 2020-12-05 | 3 |
| 41 | Male | 68 | 2020-12-08 | 0 |
| 42 | Female | 56 | 2020-12-10 | 0 |
| 43 | Female | 56 | 2020-12-16 | 0 |
| 44 | Male | 77 | 2020-12-17 | 0 |
| 45 | Male | 62 | 2020-12-07 | 3 |
| 46 | Female | 49 | 2020-12-19 | 0 |
| 47 | Male | 53 | 2020-12-19 | 2 |
| 48 | Female | 56 | 2020-12-19 | 1 |
| 49 | Male | 52 | 2020-12-25 | 1 |
| 50 | Female | 56 | 2020-12-26 | 0 |
| 51 | Male | 28 | 2020-12-26 | 0 |
| 52 | Female | 35 | 2020-12-27 | 0 |
| 53 | Male | 65 | 2020-12-27 | 1 |
| 54 | Male | 50 | 2020-12-25 | 2 |
| 55 | Female | 56 | 2020-12-26 | 3 |
| 56 | Female | 65 | 2020-12-25 | 0 |
| 57 | Female | 45 | 2020-12-28 | 0 |
| 58 | Male | 61 | 2020-12-29 | 0 |
| 59 | Male | 67 | 2021-01-02 | 0 |
| 60 | Female | 59 | 2021-01-02 | 0 |
| 61 | Male | 59 | 2021-01-05 | 0 |
| 62 | Female | 70 | 2021-01-05 | 0 |
| 63 | Female | 47 | 2021-01-06 | 0 |
| 64 | Male | 61 | 2021-01-06 | 3 |
| 65 | Male | 57 | 2021-01-06 | 1 |
| 66 | Female | 55 | 2021-01-07 | 0 |
| 67 | Male | 72 | 2021-01-07 | 1 |
| 68 | Female | 46 | 2021-01-07 | 0 |
| 69 | Male | 69 | 2021-01-07 | 0 |
| 70 | Female | 70 | 2021-01-12 | 7 |
| 71 | Male | 32 | 2021-01-12 | 0 |
| 72 | Female | 46 | 2021-01-13 | 1 |
| 73 | Female | 52 | 2021-01-13 | 6 |
| 74 | Female | 76 | 2021-01-13 | 0 |
| 75 | Male | 39 | 2021-01-13 | 0 |
| 76 | Female | 47 | 2021-01-14 | 3 |
| 77 | Male | 38 | 2021-01-14 | 0 |
| 78 | Female | 52 | 2021-01-18 | 0 |
| 79 | Male | 51 | 2021-01-18 | 8 |
| 80 | Female | 70 | 2021-01-18 | 2 |
| 81 | Male | 50 | 2021-01-20 | 0 |
| 82 | Male | 52 | 2021-01-20 | 1 |
| 83 | Female | 49 | 2021-01-26 | 2 |

**Supplementary Table 6. Gene mutations in CTCs from cancer patients**

| Sample  number | Sex | Age | Diagnosis | Stage | Pathological  mutation | CTC  count (/4mL) | Tested mutation  in CTCs | Detection time | Result |
| --- | --- | --- | --- | --- | --- | --- | --- | --- | --- |
| 1 | F | 62 | NSCLC | IV | EGFR 19del | 11 | EGFR 19del | 2020-8-8 | Positive |
| 2 | M | 65 | NSCLC | IV | EGFR 19del | 40 | EGFR 19del | 2020-8-21 | Positive |
| 3 | F | 39 | NSCLC | IV | EGFR 19del | 7 | EGFR 19del | 2020-8-3 | Positive |
| 4 | M | 40 | NSCLC | III | EGFR 19del | 3 | EGFR 19del | 2020-8-19 | Positive |
| 5 | F | 71 | NSCLC | IV | EGFR 19del | 14 | EGFR 19del | 2020-8-14 | Positive |
| 6 | F | 64 | PAAD | IV | KRAS G12D | 3 | KRAS G12D | 2020-9-3 | Negative |
| 7 | M | 62 | PAAD | IV | KRAS G12D | 5 | KRAS G12D | 2020-9-3 | Positive |
| 8 | F | 60 | PAAD | III | KRAS G12D | 25 | KRAS G12D | 2020-9-18 | Positive |
| 9 | F | 58 | PAAD | IV | KRAS G12D | 6 | KRAS G12D | 2019-11-5 | Positive |
| 10 | F | 54 | PAAD | IV | KRAS G12D | 10 | KRAS G12D | 2020-9-3 | Positive |
| 11 | F | 49 | CRC | IV | BRAF V600E | 8 | BRAF V600E | 2019-9-12 | Positive |
| 12 | M | 71 | CRC | III | BRAF V600E | 5 | BRAF V600E | 2020-7-9 | Positive |
| 13 | M | 67 | CRC | IV | BRAF V600E | 10 | BRAF V600E | 2020-9-24 | Positive |
| 14 | M | 43 | CRC | IV | BRAF V600E | 11 | BRAF V600E | 2020-4-15 | Positive |
| 15 | M | 76 | CRC | IV | BRAF V600E | 6 | BRAF V600E | 2019-12-11 | Positive |

**Supplementary Table 7. Clinical information of breast cancer patients**

| Characteristic | Number of Patients |
| --- | --- |
| **Total patient number** | 161 |
| Age at diagnosis (years) |  |
| ≤60 | 132 |
| >60 | 29 |
| Median (range) | 51 (27-76) |
| **Gender** |  |
| Male | 0 |
| Female | 161 |
| **Molecular subtype** |  |
| HER2-positive | 39 |
| Triple-negative | 35 |
| LuminalA | 12 |
| LuminalB | 5 |
| NA | 70 |
| **Stage** |  |
| Ⅰ | 40 |
| Ⅱ | 56 |
| Ⅲ | 30 |
| Ⅳ | 35 |
